# The Machine Learning Models in Major Cardiovascular Adverse Events Prediction Based on Coronary Computed Tomography Angiography: Systematic Review

**DOI:** 10.2196/68872

**Published:** 2025-06-13

**Authors:** Yuchen Ma, Mohan Li, Huiqun Wu

**Affiliations:** 1 Department of Medical Informatics Medical School of Nantong University Nantong China

**Keywords:** coronary computed tomography angiography, machine learning, major adverse cardiovascular events, radiomics, plaque

## Abstract

**Background:**

Coronary computed tomography angiography (CCTA) has emerged as the first-line noninvasive imaging test for patients at high risk of coronary artery disease (CAD). When combined with machine learning (ML), it provides more valid evidence in diagnosing major adverse cardiovascular events (MACEs). Radiomics provides informative multidimensional features that can help identify high-risk populations and can improve the diagnostic performance of CCTA. However, its role in predicting MACEs remains highly debated.

**Objective:**

We evaluated the diagnostic value of ML models constructed using radiomic features extracted from CCTA in predicting MACEs, and compared the performance of different learning algorithms and models, thereby providing clinical recommendations for the diagnosis, treatment, and prognosis of MACEs.

**Methods:**

We comprehensively searched 5 online databases, Cochrane Library, Web of Science, Elsevier, CNKI, and PubMed, up to September 10, 2024, for original studies that used ML models among patients who underwent CCTA to predict MACEs and reported clinical outcomes and endpoints related to it. Risk of bias in the ML models was assessed by the Prediction Model Risk of Bias Assessment Tool, while the radiomics quality score (RQS) was used to evaluate the methodological quality of the radiomics prediction model development and validation. We also followed the TRIPOD (Transparent Reporting of a multivariable prediction model for Individual Prognosis Or Diagnosis) guidelines to ensure transparency of ML models included. Meta-analysis was performed using Meta-DiSc software (version 1.4), which included the *I²* score and Cochran Q test, along with StataMP 17 (StataCorp) to assess heterogeneity and publication bias. Due to the high heterogeneity observed, subgroup analysis was conducted based on different model groups.

**Results:**

Ten studies were included in the analysis, 5 (50%) of which differentiated between training and testing groups, where the training set collected 17 kinds of models and the testing set gathered 26 models. The pooled area under the receiver operating characteristic (AUROC) curve for ML models predicting MACEs was 0.7879 in the training set and 0.7981 in the testing set. Logistic regression (LR), the most commonly used algorithm, achieved an AUROC of 0.8229 in the testing group and 0.7983 in the training group. Non-LR models yielded AUROCs of 0.7390 in the testing set and 0.7648 in the training set, while the random forest (RF) models reached an AUROC of 0.8444 in the training group.

**Conclusions:**

Study limitations included a limited number of studies, high heterogeneity, and the types of included studies. The performance of ML models for predicting MACEs was found to be superior to that of general models based on basic feature extraction and integration from CCTA. Specifically, LR-based ML diagnostic models demonstrated significant clinical potential, particularly when combined with clinical features, and are worth further validation through more clinical trials.

**Trial Registration:**

PROSPERO CRD42024596364; https://www.crd.york.ac.uk/PROSPERO/view/CRD42024596364

## Introduction

Coronary computed tomography angiography (CCTA) has emerged as the first-line noninvasive imaging test for patients presenting with symptoms potentially attributable to coronary artery disease (CAD), owing to its high sensitivity and specificity in diagnosing this condition [1]. Supported by a growing body of evidence and advancements in cardiac imaging technology, CCTA has solidified its role as a primary investigative tool for individuals with suspected CAD. CCTA provides comprehensive information regarding the overall coronary circulation and luminal stenosis, while also enabling the assessment of the composition, morphology, and vulnerability of atherosclerotic plaques.

Radiomics uses advanced data characterization algorithms to extract extensive quantitative information that is often imperceptible to the naked eye [2]. These radiomic features can potentially unveil tumoral patterns and provide informative multidimensional characteristics. By using radiomics to identify individuals at risk, clinicians may better select patients who could benefit from more aggressive preventive measures aimed at improving clinical outcomes in those at higher risk of CAD [3]. Radiologic images contain far more information than can be interpreted visually or quantified through simple manual measurements. This process involves extracting thousands of imaging markers from radiologic images, which describe the heterogeneity and spatial complexity of lesions. Although quantitative radiomics analysis is feasible and has the potential to enhance the diagnostic performance of CCTA, its use in predicting major adverse cardiovascular events (MACEs) remains a subject of ongoing debate [4].

A large number of radiomic features have been extracted and processed by machine learning (ML), but their stability and robustness require further validation. ML goes through the process of data preprocessing, feature extraction, analysis and selection, and predictive modeling to ultimately predict clinical outcomes. The big difference between ML and deep learning (DL) is that the latter replaces the manual feature extraction of the former with the deep extraction of specific features using pretrained models automatically, which improves the performance of the model but cannot replace the former’s interpretation of clinical practice [5]. ML models based on traditional computed tomography (CT) have been shown to enhance the long-term prediction of MACEs. ML approaches leveraging radiomics help to integrate large datasets and optimize the use of CCTA data while minimizing interpretation bias due to interobserver variability. Furthermore, radiomics-based ML analysis improves the discriminatory power of CCTA in detecting advanced atherosclerotic lesions [6].

Traditionally, MACEs encompass cardiac death (including fatal myocardial infarction [MI]), nonfatal MI (both ST-segment elevation MI and non–ST-segment elevation MI), and unstable angina requiring coronary revascularization [7]. With an increasing number of studies investigating the adverse effects of coronary artery ischemia and stenosis [8], this meta-analysis chose to include ischemia and stenosis as key indicators for the diagnostic models based on ML. Although atherosclerosis and vascular morphology significantly influenced these factors [9], they were excluded from the analysis due to their indirect association with adverse cardiac outcomes. Here, we conducted a meta-analysis and systematic review to evaluate the prognostic and diagnostic value of ML and artificial intelligence models in conjunction with CCTA-derived radiomic features. Our objective is to compare the performance of various learning algorithms and ML models, thereby providing guiding recommendations for the clinical diagnosis, treatment, and prognosis of MACEs.

## Methods

### Database Search

This meta-analysis was conducted by the PRISMA (Preferred Reporting Items for Systematic Reviews and Meta-Analyses) guidelines [10]. The checklist adhered to this study, followed by PRISMA, as shown in Table S1 in Multimedia Appendix 1. The protocol for this meta-analysis was registered with the International Prospective Register of Systematic Reviews (registration number CRD42024596364). The first submission was made on October 2, 2024, and the second revision was made on November 7, with no changes other than the title. The reason for the title change was to move the order of “ML model” forward and to emphasize one of the intervention methods, CCTA, to make the title more understandable and relevant to the full text. According to the PICO (population, intervention, control, and outcomes) framework, which are the key components of a clinical question outlined in Table S2 in Multimedia Appendix 2 [11-20] (P: Patients who underwent CCTA and were diagnosed with or predicted to develop a MACE; I: ML diagnostic or predictive models or learning algorithms; C: gold standards; O: MACE plus ischemia and stenosis), 2 independent researchers searched the Cochrane Library, Web of Science, Elsevier, CNKI, and PubMed for all available papers, with all databases searched until September 10, 2024. These researchers also recorded inclusion and exclusion reasons for these relevant papers according to the inclusion and exclusion criteria represented below, to avoid incorrect inclusion and exclusion. A third researcher resolved the dissent that occurred between the 2 independent researchers when selecting the included studies. Through discussion, keywords or phrases such as (“radiomic feature” OR “coronary plaque characteristics”) AND “CCTA[MeSH]” AND (“machine learning” OR “artificial intelligence”) AND “MACE[MeSH]” were used and searched. The full search strategies were presented in Multimedia Appendix 3. The inclusion and exclusion criteria of papers for this study can be seen in Textbox 1.

**Inclusion and exclusion criteria.**

**Inclusion criteria**

- Original research or studies containing original data.
- Prospective observational trials, case-control studies, or cohort studies.
- Research on extracting radiological features and evaluating the characteristics of coronary artery plaques using coronary computed tomography angiography (CCTA).
- Studies reporting clinical outcomes and endpoints related to major adverse cardiovascular events (MACEs) during the follow-up period.
- Studies involving registered populations that underwent ethical review or were granted an exemption from ethical review.

**Exclusion criteria**

- Studies focusing on noncoronary arteries or those that did not provide data specifically related to coronary arteries.
- Studies classified as nonoriginal research, including protocols, reviews, meta-analyses, case reports, editorials, and expert opinions.
- Studies lacking sufficient statistical information regarding effect sizes.
- Studies conducted on nonhuman participants.
- Studies published in languages other than English.
- Duplicated studies or those presenting redundant data.

### Data Extraction

The titles and abstracts were screened, and information such as the names of the first (or cofirst) authors, year of publication, type of study, type of machine algorithm, sample size of patients receiving CCTA, MACE outcomes, and radiomic features was summarized by 2 independent researchers. It was necessary to extract information to help assess the risk of bias in the study design. Any disagreements in data extraction were resolved through consultation and referral to a third researcher.

### Quality and Bias Assessments

The radiomics quality score (RQS) [21] was used to evaluate the radiomic quality of each included study to reduce bias and enhance model use in the context of radiomics. We used the Prediction Model Risk of Bias Assessment Tool (PROBAST) to assess the risk of bias and applicability concerns of each included study, using a total of 20 signaling questions in four domains—participants, predictors, outcome, and analysis [22]. TRIPOD (Transparent Reporting of a multivariable prediction model for Individual Prognosis Or Diagnosis) [23], a checklist of 22 items to validate prediction models, was used to provide more transparent details of the ML models included. Publication bias was minimized through manual searching and according to the PICO restriction. We used StataMP 17 to perform the publication bias assessment, and 2 independent researchers were involved in the quality and bias assessments. Disagreements and different judgments between reviewers were resolved by a third researcher.

### Statistical Analysis

In this meta-analysis, the pooled area under the curve (AUC) of each ML model and the forest plots were generated. Receiver operating characteristics (ROC) is the common index to draw the plots of AUC, which means the area under the receiver operating characteristic (AUROC). The standard for its diagnostic or prognostic discrimination ability was set as none (AUROC≤0.6), poor (AUROC 0.6-0.7), fair (AUROC 0.7-0.8), good (AUROC 0.8-0.9), or optimum (AUROC 0.9-1) [24]. Model calibration is a metric of goodness of fit that assesses the agreement between observed and predicted outcomes and reflects the stability of the models via calibration plots. Heterogeneity tests are categorized into drawing forest plots and hypothesis testing. The common effect measures of the former are sensitivity, specificity, etc, and the latter is the main way to achieve better testing efficacy to reduce the false negative results compared with the *G^2^* test. The *Q*-test calculated the *I^2^* statistic, which was calculated by the formula presented in



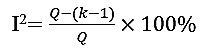



Q is the chi-square value of the heterogeneity test (χ^2^), and (*k-1*) is the degree of freedom. Usually, *I*^2^≤25% indicates low heterogeneity, moderate heterogeneity means 25%＜I^2^＜50%, and I^2^≥50% suggests high heterogeneity, which means the random effects model would be of higher efficiency. With a cutoff of 0.05 or nontraditional 0.1, a *P* value less than .05 or .10 is considered to be of high heterogeneity, and greater than .05 or .10 is considered to be of low heterogeneity, which is usually judged in conjunction with the *I^2^* value. The sample size can also have a corresponding effect on the results. For instance, if the *P* value is not significant (*P*>.05 or >.10) while the *I*^2^ value is higher than 50%, this may be due to the lack of test validity caused by the small sample size, but it does not seem to apply to our study. The high heterogeneity that occurred in our study was first chosen to be a random effects model and would be retrieved by subgroup analysis, sensitivity analysis, and meta-regression for searching heterogeneous sources and addressing potential high heterogeneity. The Meta-DiSc software program (version 1.4) [25] was used to calculate the pooled estimation of AUROC, sensitivity, specificity, positive likelihood ratio (PLR), negative likelihood ratio (NLR), and diagnostic odds ratio (DOR).

### Ethical Considerations

Since our systematic review used publicly accessible documents as evidence and therefore is no need to seek institutional ethics approval.

## Results

### Study Selection

**Figure 1:**
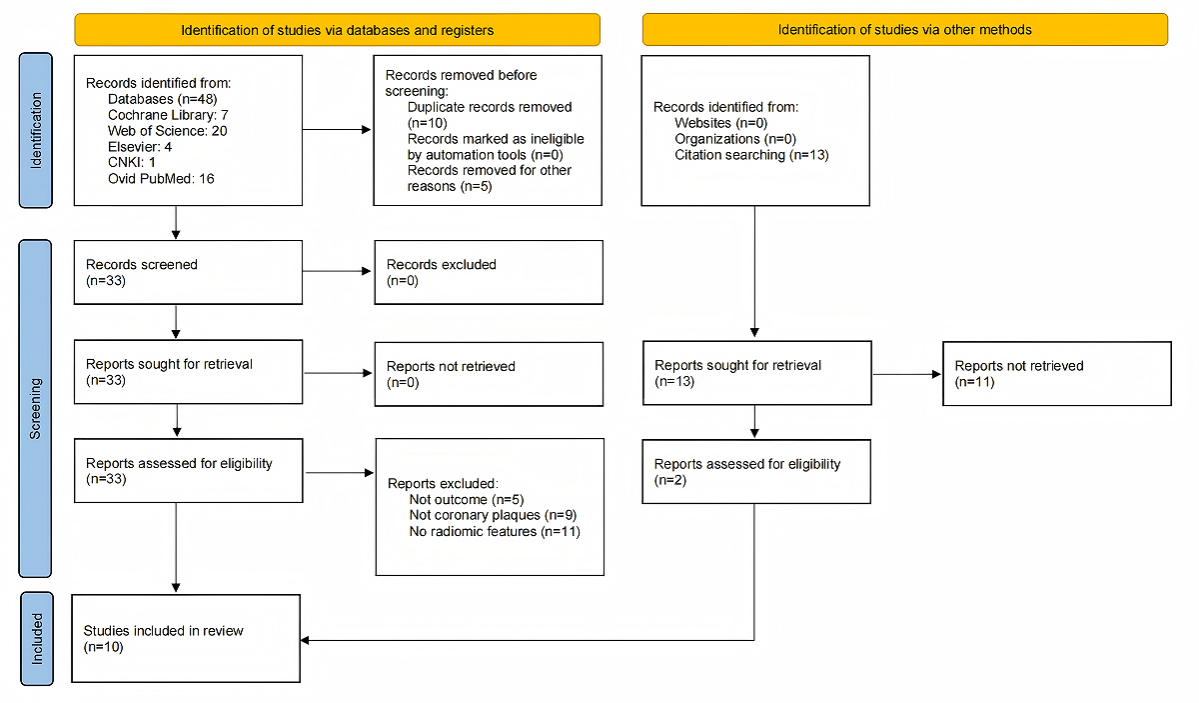
PRISMA (Preferred Reporting Items for Systematic Reviews and Meta-Analyses) flow diagram for study screening and selection. CNKI: China National Knowledge Infrastructure.

A total of 48 studies were initially identified from the Cochrane Library, Web of Science, CNKI, Elsevier, and PubMed, supplemented by 13 additional studies identified through citation searching. After removing duplicates and records that failed to meet the inclusion criteria, 33 studies underwent full screening. About 5 studies were excluded due to the lack of relevant outcomes, 9 were considered irrelevant to coronary artery plaques, and 11 were removed for lacking radiomic features. Following the consideration of 2 additional eligible citations, 10 studies [11-20] were ultimately included in this meta-analysis. The selection process, including database outputs and the deduplication process, was illustrated in the PRISMA flow diagram (Figure 1).

### Study Characteristics

The studies included in this meta-analysis were published between 2021 and 2024, with a total of 16 kinds of models identified in testing or validation groups, and 17 kinds of models found in training groups. Among the 10 studies, 5 out of 10 (50%) studies differentiated between training and testing groups with study flowcharts of inclusion and exclusion; one of these even included an additional validation group [14]. However, 1 study [18] lacked descriptions and explanations of validation or testing procedures, and thus was considered of high risk of bias [26], while 1 study [15] had 2 inner validation groups and the other had 2 external cohorts added with 1 internal cohort [19]. Most studies developed ML models at the patient level, with two exceptions [11,12]: one study [11] analyzed data at the segment level, and the second focused on the lesion level [12]. The names of the first (or cofirst) authors, year of publication, type of study, type of machine algorithm, sample size of patients included in the training group, the testing group or the validation group, MACE outcomes, and radiomic features were collected and presented in Table S3 in Multimedia Appendix 2 [11-20].

Various learning algorithms and their integrated versions were included in the analysis. Of the 10 studies, 5 used logistic regression (LR), 5 used a random forest (RF) algorithm, and 1 tested various ML algorithms to find the best one. Notably, Militello et al [18] developed L1-, tree-, and mutual information–based models, each representing different feature selection methods in 2023. Qin et al [11] implemented three algorithms: RF, LR, and artificial neural network (ANN). Overall, ML models accounted for the majority of those used, DL was applied to construct radiomic models from myocardial fibrosis features, and ANN had the best construction effect compared with RF and LR.

In the 2 studies that analyzed data at the nonpatient level, Li et al [12] in 2021 integrated 14 radiomic features and compared the RF model with a conventional LR model, achieving a sensitivity of 89.02%, a specificity of 64.91%, and an AUROC of 0.82 at the plaque level in the training set, with no statistically significant difference observed between the training set and the validation set (*P*=.58). Qin et al [11] achieved good diagnostic accuracy through the R-model (developed via multivariable LR extracting radiomic signatures) in both the training cohort (107 patients and 1712 segments) and the testing cohort (54 patients and 864 segments), achieving a sensitivity of 0.85, a specificity of 0.73, and an AUROC of 0.86.

### Quality Assessment

We used the PROBAST checklist (Table S4 in Multimedia Appendix 2 [11-20]) to assess the risk of bias and applicability of the prognostic prediction model studies. According to the criteria, the bias of participants in 2 out of 10 (20%) studies was identified as having a high risk of bias, primarily because the models in the former study lacked any external validation and the latter one did not present the concrete process or outcomes of the training group even diagnostic value. Among the 4 domains, once 1 domain was associated with a high risk of bias, the study was classified as high risk due to these significant concerns. About 5 out of 10 (50%) studies were rated as unclear, as there was at least 1 domain rated as unclear risk of bias and without high risk. The main reason for most unclear risks of bias was the lack of explicit mention of terms such as the type of study or “cohort.” Overall, 4 (40%) studies raised unclear or high concerns regarding applicability, primarily due to excessive features in the models, complicating practical data collection. The remaining studies were rated as low risk in this respect.

Publication bias could be found in Figure S1 in Multimedia Appendix 4 [11-20]. The total of 17 models in the training set (*P*=.80) and 26 models in the testing or validation set (*P*=.63) showed there was no significant bias in this meta-analysis.

The scores of each item, along with the total score for evaluating radiomic quality, were presented in Figure S2 in Multimedia Appendix 4 [11-20]. The mean RQS across studies was 17.2 (47.78%), ranging from 12 (33.33%) to 23 (63.89%). The overall performance of the included studies was fair to good; however, there were notable deficiencies, such as a lack of phantom studies across all scanners, imaging conducted at multiple time points, failure to detect and discuss biological correlates, absence of prospective study registered in a trial database, and lack of cost-effectiveness analysis. These shortcomings were not unique to this meta-analysis, and 2 independent scores from the same rater also indicated above-average reliability [27]. Moreover, moderate to high adherence was observed for most TRIPOD checklist items (Figure S3 in Multimedia Appendix 4 [11-20]), except for seven domains with poor adherence: blind assessment of outcome, blind assessment of predictors, missing data, risk groups, validation, unadjusted association between each candidate predictor and outcome, and full prediction models.

DOR is typically used as the basis for weighting each diagnostic experiment when conducting combined tests. When the DOR value is greater than 1, it indicates a positive result in diagnostic performance. Conversely, a DOR value less than 1 indicates a negative diagnostic performance. In this meta-analysis, negative diagnosis results were not considered; thus, studies with a DOR value less than 1 were excluded based on publication bias (Figure S4 in Multimedia Appendix 4 [11-20]) in both the testing group and the training group.

### Performance of ML Models for MACE Prediction

**Figure 2:**
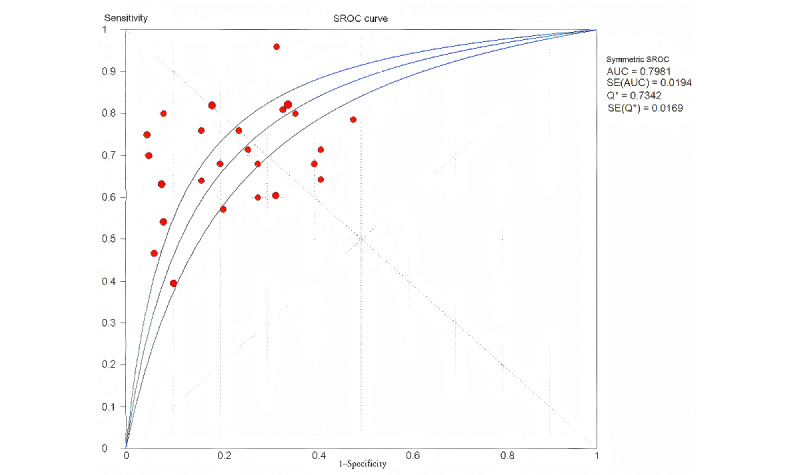
The overall pooled area under the receiver operating characteristic (AUROC) curve of machine learning models for major adverse cardiovascular events (MACE) prediction in the testing set. AUC: area under the curve; Q*: the sensitivity at the intersection of the SROC curve and the straight line (sensitivity = specificity); SROC: summary receiver operating characteristic.

**Figure 3:**
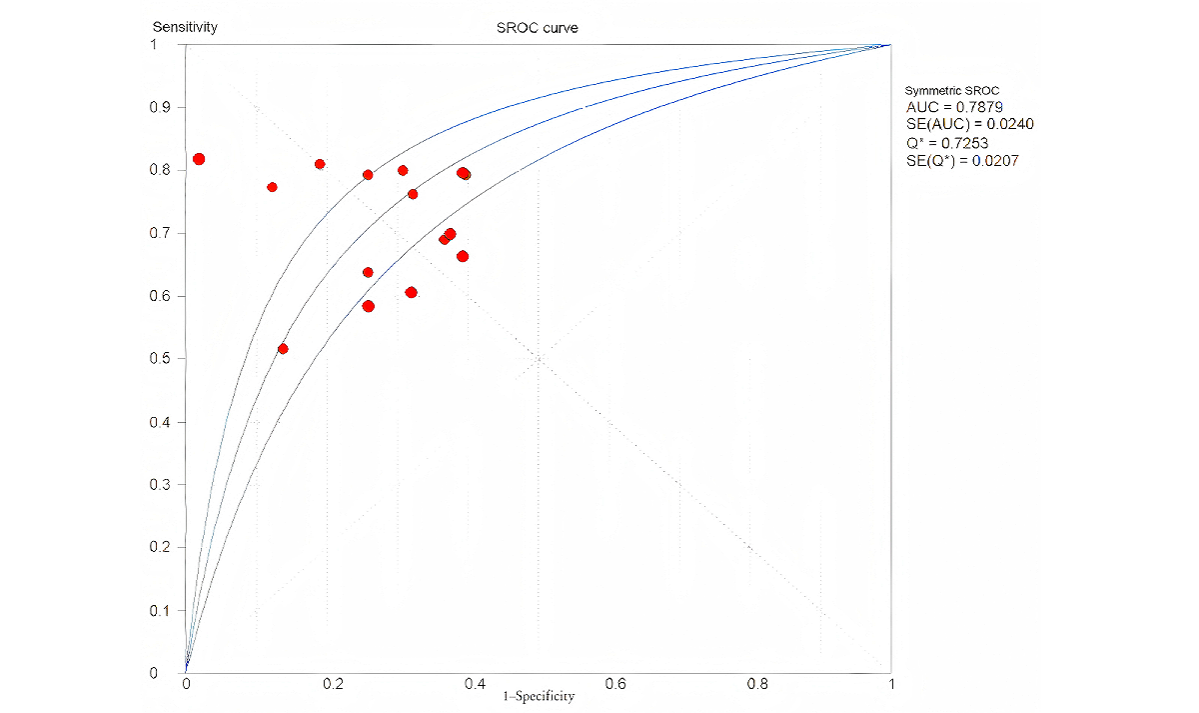
The overall pooled area under the receiver operating characteristic (AUROC) curve of machine learning models for major adverse cardiovascular events (MACE) prediction in the training set. AUC: area under the curve; Q*: the sensitivity at the intersection of the SROC curve and the straight line (sensitivity = specificity); SROC: summary receiver operating characteristic.

The overall pooled AUROC for ML models predicting MACEs was 0.7981 in the testing set (Figure 2) and 0.7879 in the training set (Figure 3). The original model evaluation metrics for each study were shown in Table S5 in Multimedia Appendix 2 [11-20]. The additional metrics were as follows.

In the training set, sensitivity was 0.71 (95% CI 0.68-0.74; *P*<.001; *I*^2^=68.3%); specificity was 0.73 (95% CI 0.70-0.75; *P*<.001; *I*^2^=88.1%); PLR was 2.57 (95% CI 2.10-3.14; *P*.<001; *I*^2^=76.1%); NLR was 0.39 (95% CI 0.33-0.47; *P*<.001; *I*^2^=71.1%); and DOR was 7.28 (95% CI 4.91-10.80; *P*<.001; *I*^2^=77.1%; Figure S5-S9 in Multimedia Appendix 4 [11-20]).

In the testing and validation set, sensitivity was 0.69 (95% CI 0.65-0.73; *P*<.001; *I*^2^=52.9%); specificity was 0.79 (95% CI 0.77-0.82; *P*<.001; *I*^2^=80.3%); PLR was 3.11 (95% CI 2.50-3.88; *P*<.001; *I*^2^=65.3%); NLR was 0.42 (95% CI 0.36-0.49; *P*=.02; *I*^2^=41.0%); and DOR was 8.71 (95% CI 6.22-12.20; *P*<.001; *I*^2^=47.5%; Figure S10-S14 in Multimedia Appendix 4 [11-20]).

### Sensitivity Analysis

Excluding the epicardial adipose tissue model (M_EAT_; AUROC 0.538, 95% CI 0.414-0.661) [13], which exhibited the lowest sensitivity and specificity with a DOR<1, the random effects meta-analysis in the training group yielded a better estimated pooled AUROC. To address the higher risk of heterogeneity, the M_EAT_ model was also excluded from the testing group. Consequently, the pooled estimates were found to be insensitive to the exclusion of outliers.

### Subgroup Analysis

Performance metrics for different ML models and MACE outcomes were examined in the subgroup analysis, in which pooled sensitivity, specificity, AUROC, DOR, PLR, and NLR were presented in Table 1. In this study, 5 included studies predicting MACEs were established using the algorithm of RF, 5 used the LR algorithm, while 3 of the 10 studies focused on ischemia during the follow-up, and 3 widely investigated MACE risks. For the different types of ML models, the LR subgroups achieved the AUROC of 0.8229 in the testing group and 0.7983 in the training group (Figure S15-S26 in Multimedia Appendix 4 [11-20]). The RF models reached an AUROC of 0.8444 in the training group (Figure S27-S32 in Multimedia Appendix 4 [11-20]), while non-LR models yielded AUROCs of 0.7390 in the testing set and 0.7648 in the training set (Figure S33-S44 in Multimedia Appendix 4 [11-20]). Moreover, the subgroup for specific MACE endpoints in the testing set produced an AUROC of 0.7343 (Figure S45-S50 in Multimedia Appendix 4 [11-20]). However, due to the limited data available from the included RF models, it was not possible to draw a direct conclusion about the predictive performance between LR and RF models in this analysis. Additionally, the tree-based model using radiomic features achieved the highest AUROC of 0.819 (SD 0.070), but when combined with clinical features using mutual information, the performance improved further to an AUROC of 0.820 (SD 0.076) [18].

**Table 1:** The pooled sensitivities, specificities, and area under the receiver operating characteristic for machine learning models predicting major adverse cardiovascular events in different subgroups.

Group and subgroup			Model, N		AUROC^a^		Sen (95% CI)^b^		Spe (95% CI)^c^		DOR (95% CI)^d^		PLR (95% CI)^e^		NLR (95% CI)^f^
**Overall**															
	Training	16		0.7879		0.71 (0.68-0.74)		0.73 (0.70-075)		7.28 (4.91-10.80)		2.57 (2.10-3.14)		0.39 (0.33-0.47)	
	Testing or validation	25		0.7981		0.69 (0.65-0.73)		0.79 (0.77-0.82)		8.71 (6.22-12.20)		3.11 (2.50-3.88)		0.42 (0.36-0.49)	
**LR^g^**															
	Training	7		0.7983		0.71 (0.67-0.75)		0.81 (0.77-0.84)		10.85 (4.58-25.74)		3.54 (2.14-5.88)		0.36 (0.25-0.52)	
	Testing or validation	18		0.8229		0.68 (0.64-0.72)		0.85 (0.82-0.87)		11.09 (7.27-16.92)		3.89 (2.92-5.20)		0.41 (0.34-0.50)	
**NLR^h^**															
	Training	9		0.7648		0.71 (0.68-0.74)		0.67 (0.64-0.70)		5.45 (3.86-7.68)		2.18 (1.86-2.55)		0.42 (0.35-0.51)	
	Testing or validation	6		0.7390		0.71 (0.63-0.78)		0.65 (0.59-0.71)		4.70 (2.96-7.44)		2.03 (1.68-2.45)		0.47 (0.36-0.61)	
**RF^i^**															
	Training	3		0.8444		0.77 (0.70-0.84)		0.74 (0.67-0.79)		10.92 (5.17-23.09)		3.10 (1.92-5.01)		0.30 (0.22-0.41)	
**MACE^j^**															
	Testing or validation	11		0.7343		0.61 (0.56-0.67)		0.85 (0.82-0.88)		8.34 (4.58-15.16)		3.81 (2.36-6.14)		0.51 (0.43-0.60)	

^a^AUROC: area under the receiver operating characteristic.

^b^Sen: sensitivity.

^c^Spe: specificity.

^d^DOR: diagnostic odds ratio.

^e^PLR: positive likelihood ratio.

^f^NLR: negative likelihood ratio.

^g^LR: logistic regression.

^h^NLR: nonlogistic regression.

^i^RF: random forest.

^j^MACE: major adverse cardiovascular event.

### Meta-Regression and Diagnostic Threshold

We performed meta-regression to address the high heterogeneity observed in subgroups. Diagnostic threshold was analyzed to address high heterogeneity and improve diagnostic accuracy, which showed no threshold effect in both the training set (Spearman correlation coefficient=–0.095; *P*=.73) and testing set (Spearman correlation coefficient=0.339; *P*=.10). The Spearman correlation coefficient was used to evaluate the correlation between certain factors occurred in this study and the calculated outcome. Generally, a coefficient greater than 0 indicated a positive correlation, while a coefficient less than 0 indicated a negative correlation. It was shown that learning algorithm, publication year, sample size, and type of ML models did not affect diagnostic accuracy (*P*>.05). The relative DOR and 95% CI were recorded in Table S6 in Multimedia Appendix 2 [11-20].

### Key Results

1. In this systematic review and meta-analysis of 10 studies, the pooled performance of ML models to diagnose MACEs had an AUROC of 0.7879 in the training set and 0.7981 in the testing set.
2. The best diagnostic value was achieved by LR in the testing or validation set, with a pooled AUROC of 0.8229, and by RF in the training set, with an AUROC of 0.8444.
3. The overall RQS assessment of the 10 included studies was moderate to good.

## Discussion

### Principal Findings

Integrating ML models based on traditional CT imaging significantly enhances the long-term prediction of MACEs and improves risk stratification [28]. In many regions, the combination of CCTA and ML has made a substantial contribution to the diagnosis of MACEs. Overall, radiomics-based ML models have shown enhanced diagnostic capabilities over traditional CCTA features in assessing coronary plaque vulnerability [29] and in differentiating between culprit and nonculprit lesions [30]. The key advantages of these approaches include their noninvasive nature and high execution rates [31]. DL methods exhibited better predictive performance in the prediction of future culprit lesions compared with basic angiographic parameters [32]. Unfortunately, very few DL models were included in the discussion of our manuscript, mainly focused on the exploration of the performance of ML models to predict MACEs. It is hoped that in the future, more DL-related model studies that meet the inclusion criteria will be included to make up for this deficiency.

According to our meta-analysis, a total of 16 models from 7 studies were included in the training group. The LR method exhibited better diagnostic performance than non-LR (AUROC 0.7983 vs 0.7648), with both categories classified as fair to good. Among non-LR methods, the RF method achieved higher sensitivity and specificity, benefiting from the diversity of radiomic features [17], with the RF algorithm constructing a radiomic signature by combining multiple features into a single value.

While the LR model demonstrates robust diagnostic performance in our study, its inherent assumptions warrant critical discussion, particularly in the biomedical context. LR assumes linear separability of classes in the feature space and a monotonic relationship between predictors and log-odds, potentially oversimplifying complex biological systems. Highly correlated inputs (making LR sensitive to multicollinearity) can distort coefficient interpretation and reduce model stability [33]. Modern artificial intelligence approaches such as attention-based neural networks or kernelized models could potentially address these limitations by learning adaptive feature representations while maintaining interpretability through techniques such as saliency mapping or Shapley value analysis [34].

The RF model, despite its second ensemble advantages in our manuscript, faces specific risks in medical applications involving high-dimensional data. When processing multiomics datasets or high-resolution medical imaging features, RF’s feature sampling mechanism may inadvertently amplify spurious correlations between noncausal variables, particularly when strong feature interdependencies exist [35]. This could lead to overfitting of training-specific noise patterns, compromising generalizability to external cohorts—a critical concern for clinical deployment.

However, the support vector machine model demonstrated lower performance, which may vary due to differences in study design or sample selection. The support vector machine radiomics model, which maximized the margin between the training patterns and the decision boundary [36], performed well in predicting vulnerable plaques with AUC values of 0.977 for the training cohort [37]. Notably, the pericoronary adipose tissue (PCAT)–model showed better sensitivity (0.80, 95% CI 0.71-0.87) compared with models focused on the 3 major epicardial coronary arteries (LAD, LCX, and RCA), while among them the RCA-model performed better specificity (0.74, 95% CI 0.65-0.82). Beyond that, the combined use of learning algorithms has increasingly become a foundational approach in modeling. Militello et al [18] found that the optimal model was the RF enhanced with mutual information, achieving an AUROC of 0.820±0.076.

Wang et al [38] used various ML models based on lesion characteristics to predict the risk of acute coronary syndrome events, finding that the XGBoost (extreme gradient boosting) model outperformed traditional methods with an AUROC of 0.918 (95% CI 0.86-0.97). Additionally, the ANN demonstrated excellent performance in predicting myocardial ischemia, achieving an AUC value of 0.721 (95% CI 0.53-0.88), using 26 radiomics features computed from CTA [39]. The integration of PCAT radiomics features based on CCTA, clinical indices, and imaging parameters into column line drawings has been proposed as a promising tool for predicting MACEs [19,40]. For instance, patients with acute MI exhibited a distinct PCAT radiomic phenotype compared with those with stable or non-CAD [40]. Consequently, radiomics-based models outperformed PCAT attenuation-based models in accurately identifying patients with MI. Beyond PCAT, radiomic nomograms also demonstrated superior performance in predicting myocardial ischemia compared with stenosis and radiomics signature alone, as they effectively identified populations at higher risk for myocardial ischemia based on myocardial segments [41].

Artificial features extracted by radiomic methods need to be processed by advanced statistical techniques or ML algorithms to ensure feature robustness and reduce data dimensionality [21,42]. This is essential for providing a unified framework for feature extraction and classification, and various challenges have emerged in the process of extracting and selecting radiomic features. For instance, usually experienced operators are assigned to perform segmentation, and most studies do not consider the variability introduced by different physicians, algorithms, software, or variations in different breathing cycles. Importantly, the combination of clinical features and radiomic features has been proven to enhance diagnostic performance compared with relying solely on either type of feature.

Some studies preprocess variables with zero variance before feature selection. To ensure repeatability and minimize redundancy, metrics such as Spearman correlation coefficient, intraclass correlation coefficient, and near-zero variance were calculated. Features with a Spearman correlation coefficient greater than 0.8 or 0.9 were excluded. Standardization using *z* score standardization, hierarchical clustering, and techniques such as the least absolute shrinkage and selection operator and the maximum relevance minimum redundancy were subsequently applied to further refine the feature set. In our study, 2 studies constructed radiomic signatures to consolidate multiple features into a single value, and another 2 studies used rad-score to evaluate combined or individual models. Huang et al [19] used 4 supervised methods for irrelevancy reduction and 6 unsupervised methods for redundancy reduction. Militello et al [18] considered 3 feature selection methods: L1-, tree-, and mutual information–based.

Predictive or diagnostic ML models have used various plaque features, including minimum lumen area, luminal stenosis, percent atheroma volume, fibrous tissue, fibrotic fatty tissue, the Napkin-ring (NR) sign, necrotic core, calcium content, lesion length, plaque volume, proximal left anterior descending coronary artery lesion, and remodeling index [38,43-45]. Among these, the classification of noncalcified plaques is particularly critical in predicting cardiac events [46]. High-risk plaques (HRPs) identified by CCTA, as vital factors in identifying future risk of MACEs in the early period, are typically characterized by plaque burden ≥70%, minimum lumen area <4 mm², low-attenuation plaque, and positive remodeling, all of which have been independently associated with cardiovascular events [47]. Among those, low-attenuation plaque ≤60 Hounsfield unit and NR sign are the most powerful HRP-independent predictors for MACEs. In our manuscript, 1 of 10 adopted HRP into patients’ basic clinical data, corroborating evidence that HRP is an important factor in the pathogenesis of MACEs and has maximum use in the detection of CCTA. Li et al [12] in 2021 reported 4 vulnerable characteristics, including noncalcified plaque volume, NR sign, remodeling index, and spotty calcification, and claimed that compared with traditional plaque assessment, plaque radiomics analysis had greatly improved in identifying hemodynamically significant coronary stenosis.

Despite the radiomic features collected and discussed above, other important features should also be taken into consideration to predict MACEs. The index of microvascular resistance of the coronary artery, evaluating coronary microvascular dysfunction, can even lead to perioperative myocardial injury [48]. Nowadays, a novel and upgrading angiographically derived index of microcirculatory resistance was reported to be associated with better prediction of MACEs after percutaneous coronary intervention, with the best cutoff value of 25.17U to diagnose perioperative myocardial injury [49]. Fractional flow reserve (FFR), one of the hemodynamic parameters, especially using CCTA, namely FFRCT, played an important role in assessing and detecting stenosis of coronary arteries. Usually, an FFRCT<0.8 was considered a positive index, and a lower FFRCT score caused a higher risk for MACEs [50]. One study reported that measurement of CT-FFR 1 to 2 centimeters distal to the target lesion helped to avoid unnecessary injury while preserving diagnostic efficacy, and CT-FFR 1/2cm has been shown to provide better risk stratification for future adverse cardiac events [51]. It was also generally accepted that the clinical use and diagnostic results of FFRCT were independent of age [52]. The combined application of ML added a higher predictive value to FFRCT and provided a new research tool for future noninvasive diagnosis and treatment [53]. High-sensitivity C-reactive protein (hs-CRP), commonly representing the development of systemic inflammation, deeply interacted with the triglyceride glucose index, and its growing number suggested a higher risk of MACEs and coronary occlusion [54,55]. Kraaijenhof et al [56] in 2025 found that after 20 years of follow-up, the multivariable-adjusted hazard for hs-CRP predicting MACEs was 1.55 (95% CI 1.37-1.74). Both FFR and hs-CRP were considered as strong independent predictors of MACEs and CAD [57,58], and we hoped they could be included in our future follow-up studies. As a protective factor, low left ventricular ejection fraction was associated with high T1 (>1250 ms) in noninfarcted myocardium, which was an independent predictor of MACEs [59]. Patients with severely reduced left ventricular ejection fraction were recommended to undergo coronary artery bypass grafting rather than percutaneous coronary intervention to avoid higher mortality and MACE rates [60].

However, different voices are pointing toward radiomic features. Huang et al [42] designed a framework with a two-stream convolutional neural network feature extraction module to extract the radiomic features of CAD plaques. This study was conducted based on the limitations of radiomics methods in extracting features of PCAT and atherosclerotic plaques. The limitations were due to the limited artificially extracted features and the excessively complex statistical or ML methods for feature processing.

Class imbalance and imbalanced set ratio are worth noting in our manuscript. It has been reported that the precision-recall curve is more informative and powerful for imbalanced cases than ROC with more accurate classifier performance [61]. Though ROC plots are more widely used in binary classifiers, class imbalance may mislead the ROC plots to some degree. All 10 studies included in this study adopted the AUROC plots, without avoiding the bias caused by class imbalance. Moreover, another reason for model overfitting is the imbalance ratio between the training and testing groups. The most suitable ratio for LR models is expected to be 9:1, while high accuracy can still be achieved even when the testing set data is very small in RF models [62].

In addition to predictive performance, the use of ML algorithms plays a crucial role in their adoption and application in real-world scenarios. Many previously proposed models required users to write complex code, perform manual parameter tuning, or configure appropriate environments in specific programming languages, which could pose significant barriers for nonexpert users. Some algorithms even lacked user-friendly interfaces or did not offer software packages or online tools, further limiting accessibility. Therefore, the ML model is expected to develop into an independent software application or an internet-based web server, which will significantly enhance use by lowering the technical threshold for end users and thus become a practical tool [63]. Zhou et al [64] in 2023 established an intelligent diagnostic platform by using radiomics and a convolutional neural network–based DL algorithm, and it was proved to have high functionality and efficiency. This provides great guiding significance for the further development of our research, improving the prediction accuracy of MACEs by lowering the threshold for constructing a model combining radiomics and ML.

### Limitations

Our study had several unavoidable limitations. First, despite comprehensive screening, we included only 10 original studies, which limited our ability to conduct various subgroup analyses on specific MACE endpoints and different plaque features. Second, high heterogeneity presented a significant constraint. Both the training and testing sets exhibited moderate to high heterogeneity, with the overall heterogeneity in the testing group being lower than that in the training group. Variations in sample sizes, execution settings, and selected ML models across different studies impacted the final results, particularly considering the differing ML algorithms used for diagnosis and the various feature selection methods used. Future research that incorporates more studies focusing on specific MACE endpoint subgroups, diverse ML algorithms, various plaque features, and extended follow-up periods may provide stronger evidence.

Our evaluation using the RQS indicated that the included studies demonstrated a high quality of scientific practice. Finally, we combined predictive and diagnostic ML models in our analysis because they shared a similar process for deriving conclusions about MACEs from radiomic features obtained via CCTA. It is important to note that the predictive models included in our analysis were retrospective studies, assessing outcomes from a more distant point in time to a more recent one, and the MACE endpoints they predicted also occurred in the past. Therefore, while these 2 types of models are fundamentally different, we chose to combine them to achieve a more comprehensive and complete result, supported by meta-regression analysis (*P*=.08 in the training group; *P*=.10 in the testing group).

### Clinical Implications

Clinical studies found that the hybrid ML models outperformed most LR models and traditional CCTA scoring systems, especially RF. The key consideration was which features should be selected for the model’s variables, both clinical and anatomical, which would greatly affect its efficacy. By incorporating ML into clinical practice, health care professionals can significantly improve the risk stratification of patients with CAD. Second, the implementation of ML models will lead to more personalized clinical treatment strategies. In addition, the adoption of ML-based tools in diagnostic workflows can simplify clinical decision-making.

However, introducing ML models into clinical practice is not without challenges. It requires the integration of high-quality annotated datasets and must ensure that the models are interpretable to gain the trust of clinicians. Moreover, models must be validated in different patient populations and clinical environments to ensure that they will not be confounded by the complexities of real-world scenarios that can reduce their efficacy.

### Conclusion

The application of ML models to coronary plaque features based on CCTA for the prediction of MACEs offers a promising avenue for improving cardiovascular care. The diagnostic value of ML models for predicting MACEs has been proven to be superior to general models based on basic feature extraction and integration from CCTA. In particular, LR-based ML diagnostic models hold significant clinical promise when combined with clinical features, and more clinical trial studies are needed for validation.

## Data Availability

The datasets used and analyzed during the current study are available from the corresponding author on reasonable request.

## Appendix

Multimedia Appendix 1 PRISMA (Preferred Reporting Items for Systematic Reviews and Meta-Analyses) checklist.

Multimedia Appendix 2 Additional tables for this meta-analysis.

Multimedia Appendix 3 The full searching strategies.

Multimedia Appendix 4 Additional figures for this meta-analysis.

## Abbreviations

ANN: artificial neural network
AUC: area under the curve
AUROC: area under the receiver operating characteristic
CAD: coronary artery disease
CCTA: coronary computed tomography angiography
CT: computed tomography
DL: deep learning
DOR: diagnostic odds ratio
FFR: fractional flow reserve
HRP: high-risk plaque
hs-CRP: high-sensitivity C-reactive protein
LR: logistic regression
MACE: major adverse cardiovascular event
MEAT: epicardial adipose tissue model
MI: myocardial infarction
ML: machine learning
NLR: negative likelihood ratio
NR: napkin-ring
PCAT: peri-coronary adipose tissue
PICO: population, intervention, control, and outcomes
PLR: positive likelihood ratio
PRISMA: Preferred Reporting Items for Systematic Reviews and Meta-Analyses
PROBAST: Prediction model Risk Of Bias Assessment Tool
RF: random forest
ROC: receiver operating characteristic
RQS: radiomics quality score
TRIPOD: Transparent Reporting of a multivariable prediction model for Individual Prognosis Or Diagnosis
XGBoost: extreme gradient boosting
