# Supplementary material for: The Machine Learning Models in Major Cardiovascular Adverse Events Prediction Based on Coronary Computed Tomography Angiography: Systematic Review
Source: J Med Internet Res. 2025 Jun 13;27:e68872. doi: 10.2196/68872 (PMC12205263; doi:10.2196/68872)
Supplement: Multimedia Appendix 2 [file jmir_v27i1e68872_app2.docx]

Multimedia Appendix 2

**Table S2.** Selection criteria of predictive modelling studies in PICOTS format

|  | **Participants**  **(P)** | **Intervention**  **(I)** | **Control (C)** | **Outcomes**  **(O)** | **Timeframe**  **(T)** | **Setting**  **(S)** | **Other limits** |
| --- | --- | --- | --- | --- | --- | --- | --- |
| **Inclusion criteria** | Patients underwent CCTA and diagnosed or predicted MACE | ML diagnostic or predictive models;  Learning algorithms | Golden standards | MACE plus ischemia and stenosis | Up to September, 10, 2024 | **Clinical care settings:**  Such as hospitals, institutions and so on  **type :**  Retrospective  studies | Language:  English |
| **Exclusion criteria** | Patients underwent other angiograms | Radiomic features were excluded;  Non-ML models;  No clear ML algorithms |  |  |  |  | Other language |

**Table S3.** Study characteristics

| Study | Population | | | Study  Design | MACE  Outcome | Machine  Model | Model evaluation metrics | Radiomics  Feature | | |
| --- | --- | --- | --- | --- | --- | --- | --- | --- | --- | --- |
|  | Training | Testing | Validation |  |  |  |  |  |  |  |
| Feng,  2022 | 134 | 150 | 66 | Retrospective | functional ischemia | Boruta integrating with a random forest algorithm | AUC,ACC,  SEN,SPE | From the 47 features highly relevant to functional ischemia, the top-8 contributive features were selected to establish Rad-signature | log.sigma.2.5.mm.3D_firstorder_Median,  wavelet.LLH_gidm_DependenceEntropy,  original_shape_Maximum2DDiameterSlice ,  log.sigma.0.5.mm.3D_firstorder_Skewness,  wavelet.LLH_glcm_Imc1,  exponential_firstorder_Minimum,  wavelet.LLH_gldm_LowGrayLevelEmphasis,  square_glcm_SumSquares | |
| Jing,  2024 | 261 | 132 | NA | Retrospective  cohort | acute coronary syndrome | a fully automatic workflow--- models AC1, AC2, and AC3，models AN1, AN2, and AN3  (LR) | AUC,ACC,SEN,SPEPPV,NPV | 94 features from the three main branches (LAD, LCX, RCA),  including 19 first-order and 75 texture features, yielding a total of 282 features for all three arteries | NA | |
| Li,  2021 | 118 patients with  139  plaques | NA | 31  patients with  35  plaques | Retrospective  cohort | hemodynamically significant coronary stenosis | the random forest (RF) | ACC,SEN,SPE,  PPV,NPV | NCP volume, NRS, remodeling index, and spotty calcification were included in  the conventional model and 14 features were integrated to build the radiomics model | logarithm_gldm_GrayLevelNonUniformity,  square_glcm_Imc1,  wavelet-LLH_firstorder_Kurtosis,  wavelet-HLL_ngtdm_Strength,  logarithm_ngtdm_Strength,  wavelet-HLL_glcm_DifferenceVariance,  square_glszm_GrayLevelVariance,  wavelet-LHL_firstorder_MeanAbsoluteDeviation,  logarithm_gldm_DependenceEntropy,  wavelet-HLH_gldm_GrayLevelNonUniformity,  log-sigma-0-5-mm-3D_glcm_InverseVariance ,  log-sigma-0-5-mm-3D_gldm_GrayLevelNonUniformity,  wavelet-LLL_gldm_SmallDependenceEmphasis,  wavelet-LLL_firstorder_Kurtosis | |
| Militello,2023 | 118 | NA | NA | Retrospective | Coronary  Artery Disease | SVM, Random  Forest, AdaBoost and XGBoost | AUC,ACC,SEN,SPEPPV,NPV | 93 radiomics features were extracted for each ROI around the IVA | L1-  based | Age,10Percentile,Mean,Minimum,  DependenceNonUniformity,  LargeDependenceHighGrayLevelEmphasis,  LargeDependenceLowGrayLevelEmphasis,  GrayLevelNonUniformity,  SizeZoneNonUniformity,Busyness |
|  |  |  |  |  |  |  |  |  | Tree-  based | Age,current hypertension,statin treatment  Vasculopathy,10Percentile,90Percentile  Energy,Mean,TotalEnergy,ClusterShade  LargeDependenceHighGrayLevelEmphasis  LargeDependenceLowGrayLevelEmphasis  GrayLevelNonUniformity  GrayLevelNonUniformityNormalized  GrayLevelVariance,LowGrayLevelZoneEmphasis,SizeZoneNon Uniformity,SmallAreaHighGrayLevelEmphasis SmallAreaLowGrayLevelEmphasis  ZoneEntropy |
|  |  |  |  |  |  |  |  |  | Mutual Information | Age,10Percentile,Energy,TotalEnergy  Autocorrelation,GrayLevelNonUniformity,  GrayLevelNon, UniformityNormalized,  GrayLevel Variance,  SizeZoneNon UniformityNormalized,  Busyness |
| Qin,  2021 | 107 patients  with 1712 segments | 54 patients with 864 segments | NA | Retrospective  cohort | myocardial fibrosis in hypertrophic cardiomyopathy | Rad-sig (R-model), clinical characteristic (C-model)  (random forest, least absolute shrinkage and selection opera tor with logistic regression, and artificial neural network (ANN)) | AUC,ACC,SEN,SPE | 28 features were shown ranked importance in the  model by the random forest based on the Gini-impurity and utilized to build R-model and generate Rad-sig | wavelet.LHL_glcm_SumEntropy ,wavelet.LHL_firstorder,  Maximumwavelet.LLL_gicm_ClusterProminence,  wavelet.LLH_firstorder_Skewness,  wavelet.HHH_firstorder_InterquartileRange,  original_giszm_LowGrayLevelZoneEmphasis, wavelet.HLH_glszm_HighGrayLevelZoneEmphasis,  logarithm_gicm_ld  (＞0.05) | |
| Wang,  2023 | 116 | NA | 50;50  (internal) | Retrospective  cohort | non-ST-segment elevation myocardial  infarction | EAT radiomics model  (LR) | AUC,ACC,SEN,SPEPPV,NPV | Eight radiomics features of EAT, sixteen of RCA-PCAT, fifteen of LAD-PCAT, and eighteen  of LCX-PCAT | NA | |
| Wang,  2024 | 110 | 48 | NA | Retrospective | myocardial ischemia | a random forest model---- hybrid machine learning (HML) model | AUC,ACC,SEN,SPE | logarithm_ngtdm_Strength (LNS), waveletLLH_glcm_MCC (WLGM), and gradient_firstorder_Energy (GFE) from1,316 radiomics features | NA | |
| You,  2023 | 201 | NA | 87 | Retrospective  cohort | MACE risks | PCAT, EAT, clinical, PCAT-clinical, and EAT-clinical mode  （LR） | AUC,ACC,SEN,SPEPPV,NPV | A total of 184 radiomics features were extracted from EAT and PCAT images | 12 significant radiomics features were retained to generate  PCAT-score and 2 were retained for the EAT-score. | |
| Zhang,  2024 | 225 | 57 | NA | Retrospective  cohort | MACE | LAD-Mode,LCX-Model,RCA-Model,PCAT-Model ,Cli-Mode,Overall Model  （SVM） | AUC,ACC,SEN,SPEPPV,NPV | 38 features from the RCA, 34 features from the LAD, 34 features from the LCX, and 31  features from the PCAT |  | |
| Huang,  2024 | 192 | NA | 82  (Internal)  117,87  (External) | Retrospective  cohort | MACE | Clinical model, radiological model, radiomics model, and the integrated model  （LR） | AUC,ACC,SEN,SPEPPV,NPV | three first-order statistical features, two morphological features, and seven textural features | original_shape_LeastAxisLength,  wavelet-HHL_glszm_GrayLevelNonUniformity,  wavelet-HLH_glszm_ZoneEntropy ,  wavelet-LLL_ngtdm_Coarseness,  original_shape_Maximum2DDiameterColumn,  wavelet-LLH_girim_LongRunHighGrayLevelEmphasis,  wavelet-LHL_firstorder_Maximum,  wavelet-LHL_glcm_idn,  wavelet-LLH_firstorder_Maximum ,  wavelet-LHL_gidm_GrayLevelNonUniformity,  wavelet-HLL_firstorder_TotalEnergy,  wavelet-LLL_giszm_SmallAreaLowGrayLevelEmphasis | |

**Table S4.** Risk of bias and applicability assessment by PROBAST criteria

| Study | group | ROB | | | | Overall bias rating | Overall applicability rating |
| --- | --- | --- | --- | --- | --- | --- | --- |
|  |  | Participants | Predictors | Outcome | Analysis |  |  |
| Feng,2022 | dev | low | low | low | low | low | low |
|  | val | low | low | low | low |  |  |
| Jing,2024 | dev | low | unclear | low | low | unclear | low |
|  | val | low | unclear | low | low |  |  |
| Li,2021 | dev | low | low | low | low | low | low |
|  | val | low | low | low | low |  |  |
| Militello,2023 | dev | unclear | unclear | low | low | high | high |
|  | val |  |  |  |  |  |  |
| Qin,2021 | dev | low | low | low | low | low | low |
|  | val | low | low | low | low |  |  |
| Wang,2023 | dev | low | unclear | unclear | low | low | unclear |
|  | val | low | unclear | unclear | low |  |  |
| Wang,2024 | dev | unclear | unclear | low | low | low | unclear |
|  | val | unclear | unclear | low | low |  |  |
| You,2023 | dev | low | low | unclear | low | unclear | low |
|  | val | low | low | unclear | low |  |  |
| Zhang,2024 | dev | low | unclear | low | low | unclear | low |
|  | val | low | unclear | low | low |  |  |
| Huang,2024 | dev | low | high | unclear | unclear | high | high |
|  | val | low | high | low | low |  |  |

* When a single study included multiple models, risk of bias and applicability concerns were assessed for each model. Overall ratings per study have been presented since any discrepancies or counter-intuitive case scenarios with contradictory ratings between models within any single study were not observed.

**Table S5.** original Model evaluation metrics for each study on patient-level

| Study | Model | Group type | | AUC^a^(95%CI^b^) | ACC(95%CI) ^c^ | Sen ^d^ | Spe ^e^ | PPV ^f^ | NPV ^g^ | |
| --- | --- | --- | --- | --- | --- | --- | --- | --- | --- | --- |
| Feng,2022 | Rad-signature | Training | | 0.83 (0.78–0.87) | 0.72 (0.67–0.76) | 0.80 | 0.69 | NA | NA | |
|  |  | Validation | | 0.82 (0.74–0.89) | 0.71 (0.64–0.77) | 0.81 | 0.67 | NA | NA | |
|  |  | Testing | | 0.82 (0.77–0.86) | 0.69 (0.64–0.73) | 0.82 | 0.66 | NA | NA | |
| Jing,2024 | AN3(LR) | Training | | 0.94 (0.879-1.002) | 0.92 (0.86-0.95) | 0.82 | 0.98 | 0.964 | 0.898 | |
|  |  | Testing | | 0.89 (0.795-0.991) | 0.82 (0.71-0.91) | 0.82 | 0.82 | 0.737 | 0.885 | |
| Militello,2023 | radiomic | Training | L1-based | 0.741 ± 0.081 | 0.659 ± 0.077 | 0.691 ± 0.112 | 0.635 ± 0.126 | 0.656 ± 0.115 | 0.673 ± 0.113 | |
|  |  |  | Tree-based | 0.819 ± 0.074 | 0.720 ± 0.078 | 0.767 ± 0.112 | 0.681 ± 0.130 | 0.709 ± 0.112 | 0.747 ± 0.118 | |
|  |  |  | Mutual Information | 0.803 ± 0.076 | 0.713 ± 0.078 | 0.762 ± 0.112 | 0.672 ± 0.124 | 0.700 ± 0.112 | 0.740 ± 0.118 | |
| Wang,2023 | EAT | Training | | 0.708  (0.614–0.802) | 0.689 | 0.637 | 0.741 | 0.711 | | 0.671 |
|  |  | internal validation 1 | | 0.693  (0.546–0.840) | 0.660 | 0.720 | 0.681 | 0.642 | | 0.638 |
|  |  | internal validation 2 | | 0.691  (0.535–0.847) | 0.720 | 0.800 | 0.640 | 0.690 | | 0.762 |
|  | RCA-PCAT | Training | | 0.833 (0.759–0.906) | 0.767 | 0.793 | 0.741 | 0.754 | | 0.781 |
|  |  | internal validation 1 | | 0.837 (0.729–0.945) | 0.740 | 0.640 | 0.840 | 0.800 | | 0.700 |
|  |  | internal validation 2 | | 0.822 (0.701–0.944) | 0.800 | 0.760 | 0.840 | 0.826 | | 0.778 |
|  | LAD-PCAT | Training | | 0.720 (0.628–0.813) | 0.689 | 0.517 | 0.862 | 0.789 | | 0.641 |
|  |  | internal validation 1 | | 0.766 (0.625–0.907) | 0.760 | 0.760 | 0.760 | 0.760 | | 0.760 |
|  |  | internal validation 2 | | 0.760 (0.621–0.899) | 0.740 | 0.680 | 0.800 | 0.773 | | 0.714 |
|  | LCX-PCAT | Training | | 0.713 (0.619–0.807) | 0.698 | 0.793 | 0.603 | 0.666 | | 0.744 |
|  |  | internal validation 1 | | 0.675 (0.521–0.829) | 0.640 | 0.680 | 0.600 | 0.629 | | 0.652 |
|  |  | internal validation 2 | | 0.674 (0.517–0.830) | 0.700 | 0.680 | 0.720 | 0.708 | | 0.692 |
|  | Combined | Training | | 0.889 (0.832–0.946) | 0.810 | 0.810 | 0.810 | 0.810 | 0.810 | |
|  |  | internal validation 1 | | 0.898 (0.802–0.993) | 0.860 | 0.800 | 0.920 | 0.909 | | 0.821 |
|  |  | internal validation 2 | | 0.866 (0.769–0.963) | 0.820 | 0.960 | 0.680 | 0.750 | 0.944 | |
| Wang,2024 | Radiomics | Training | | 0.906 (0.851–0.961) | 0.845 (0.843–0.848) | 0.776 | 0.902 | NA | NA | |
|  |  | Testing | | 0.831 (0.719–0.943) | 0.729 (0.721–0.737) | 0.714 | 0.741 | NA | NA | |
| You, 2023 | PCAT | Training | | 0.690  (0.616, 0.763) | 0.642 | 0.604 | 0.680 | 0.656 | 0.630 | |
|  |  | Validation | | 0.703  (0.591, 0.816) | 0.644 | 0.605 | 0.682 | 0.650 | 0.638 | |
|  | EAT | Training | | 0.543  (0.463, 0.623) | 0.502 | 0.446 | 0.560 | 0.506 | 0.500 | |
|  |  | Validation | | 0.538  (0.414, 0.661) | 0.482 | 0.395 | 0.568 | 0.472 | 0.490 | |
| Zhang,2024 | LAD | Training | | 0.679  (0.609,0.748) | 0.662 | 0.699 | 0.625 | 0.653 | 0.673 | |
|  |  | Testing | | 0.664  (0.517,0.811) | 0.649 | 0.785 | 0.517 | 0.611 | 0.714 | |
|  | LCX | Training | | 0.651  (0.580,0.723) | 0.636 | 0.664 | 0.607 | 0.630 | 0.642 | |
|  |  | Testing | | 0.623 (0.474, 0.773) | 0.614 | 0.643 | 0.586 | 0.600 | 0.630 | |
|  | RCA | Training | | 0.706 (0.638, 0.773) | 0.662 | 0.584 | 0.741 | 0.695 | 0.639 | |
|  |  | Testing | | 0.675 (0.531, 0.820) | 0.684 | 0.571 | 0.793 | 0.727 | 0.657 | |
|  | PCAT | Training | | 0.764 (0.703, 0.825) | 0.702 | 0.797 | 0.607 | 0.672 | 0.747 | |
|  |  | Testing | | 0.723 (0.589, 0.857) | 0.649 | 0.714 | 0.586 | 0.625 | 0.680 | |
| Huang,2024 | Radiological | Internal validation | | 0.887 | 0.891 | 0.7 | 0.944 | 0.918 | 0.778 | |
|  |  | External validation 1 | | 0.829 | 0.861 | 0.632 | 0.924 | 0.839 | 0.8 | |
|  |  | External validation 2 | | 0.817 | 0.825 | 0.55 | 0.922 | 0.839 | 0.733 | |
|  | Radiomics | Internal validation | | 0.901 | 0.854 | 0.481 | 0.941 | 0.886 | 0.658 | |
|  |  | External validation 1 | | 0.735 | 0.833 | 0.395 | 0.899 | 0.755 | 0.652 | |
|  |  | External validation 2 | | 0.901 | 0.943 | 0.75 | 0.961 | 0.907 | 0.882 | |

^a^ AUC: area under the ROC curve.

^b^ CI: confidence interval. Some studies used (mean ± stdDev) to evaluate would also be recorded in this table.

^c^ ACC, accuracy.

^d^ Sen, sensitivity.

^e^ Spe, specificity.

^f^ PPV, positive predictive value.

^g^ NPV, negative predictive value.

**Table S6.** Meta-regression and analysis of diagnostic threshold

**Training Group**

**Meta-Regression(Inverse Variance weights)**

Var Coeff. Std. Err. p - value RDOR [95%CI]

-----------------------------------------------------------------------------------------

Cte. 3.215 0.4829 0.0001 ---- ----

S -0.215 0.2528 0.4140 ---- ----

LR/NLR 0.560 0.5150 0.3024 1.75 (0.56;5.52)

Year -0.159 0.8260 0.8515 0.85 (0.14;5.38)

Sample size -0.204 0.6296 0.7523 0.82 (0.20;3.32)

Model -1.558 0.7987 0.0798 0.21 (0.04;1.25)

-----------------------------------------------------------------------------------------

Tau-squared estimate = 0.2864 (Convergence is achieved after 7 iterations)

Restricted Maximum Likelihood estimation (REML)

No. studies = 16

Filter OFF

Add 1/2 to all cells of the studies with zero

**Analysis of Diagnostic Threshold**

-------------------------------------------------------------------------------

Spearman correlation coefficient: -0.095 p-value= 0.726

(Logit(TPR) vs Logit(FPR)

--------------------------------------------------------------------------------

Moses' model (D = a + bS)

Weighted regression (Inverse Variance)

Var Coeff. Std. Error T p-value

--------------------------------------------------------------------------------

a 1.942 0.207 9.367 0.0000

b(1) -0.601 0.243 2.469 0.0270

--------------------------------------------------------------------------------

Tau-squared estimate = 0.5152 (Convergence is achieved after 6 iterations)

Restricted Maximum Likelihood estimation (REML)

No. studies = 16

Filter OFF

Add 1/2 to all cells of the studies with zero

**Testing Group**

**Meta-Regression(Inverse Variance weights)**

Var Coeff. Std. Err.p - value RDOR [95%CI]

--------------------------------------------------------------------------------------Cte. 2.085 0.4143 0.0001 ---- ----

S 0.009 0.1906 0.9639 ---- ----

LR/NLR 1.753 0.6273 0.0116 5.77 (1.55;21.45)

Year -1.139 0.5822 0.0652 0.32 (0.09;1.08)

Samplesize -0.440 0.4447 0.3348 0.64 (0.25;1.63)

Model -0.667 0.4532 0.1573 0.51 (0.20;1.32)

--------------------------------------------------------------------------------------

Tau-squared estimate = 0.0656 (Convergence is achieved after 6 iterations)

Restricted Maximum Likelihood estimation (REML)

No. studies = 25

Filter OFF

Add 1/2 to all cells of the studies with zero

**Analysis of Diagnostic Threshold**

--------------------------------------------------------------------------------

Spearman correlation coefficient: 0.339 p-value= 0.097

(Logit(TPR) vs Logit(FPR)

--------------------------------------------------------------------------------

Moses' model (D = a + bS)

Weighted regression (Inverse Variance)

Var Coeff. Std. Error T p-value

--------------------------------------------------------------------------------

a 2.032 0.173 11.714 0.0000

b(1) -0.244 0.133 1.843 0.0782

--------------------------------------------------------------------------------

Tau-squared estimate = 0.2509 (Convergence is achieved after 4 iterations)

Restricted Maximum Likelihood estimation (REML)

No. studies = 25

Filter OFF

Add 1/2 to all cells of the studies with zero
