# Supplementary material for: The Machine Learning Models in Major Cardiovascular Adverse Events Prediction Based on Coronary Computed Tomography Angiography: Systematic Review
Source: J Med Internet Res. 2025 Jun 13;27:e68872. doi: 10.2196/68872 (PMC12205263; doi:10.2196/68872)
Supplement: Multimedia Appendix 3 [file jmir_v27i1e68872_app3.doc]

**Supplement File 1**

| Supplement file 1 Search strategies for all databases | |
| --- | --- |
| Database | Search strategy |
| PubMed | (Radiom*) AND (Coronary artery disease[MeSH Terms] OR CAD[MeSH Terms] OR Coronary Disease[MeSH Terms] OR Coronary Atherosclerosis[MeSH Terms] ) AND MACE[MeSH Terms] OR major adverse cardiovascular events[MeSH Terms] AND (CT[MeSH Terms] OR coronary CT angiography[MeSH Terms] OR coronary computed tomographic angiography[MeSH Terms] OR CCTA[MeSH Terms] AND machine learning[All Fields] OR artificial intelligence[All Fields] OR AI[All Fields] OR deep learning [All Fields] OR learning algorithm[All Fields] ) |
| Web of Science | (Radiom*(AB)) AND AND (Coronary artery disease(AB) OR CAD(AB) OR Coronary Disease(AB) OR Coronary Atherosclerosis(AB) ) AND (MACE(AB)OR major adverse cardiovascular events(AB)) AND AND ((CT(AB) OR coronary CT angiography(AB) OR coronary computed tomographic angiography(AB) OR CCTA(AB)) AND machine learning(AB) OR artificial intelligence(AB) OR AI(AB) OR deep learning (AB) OR learning algorithm(AB) . |
| The Cochrane Library | (Radiom*) AND (Coronary artery disease[MeSH Terms] OR CAD[MeSH Terms] OR Coronary Disease[MeSH Terms] OR Coronary Atherosclerosis[MeSH Terms] ) AND MACE[MeSH Terms] OR major adverse cardiovascular events[MeSH Terms] AND (CT[MeSH Terms] OR coronary CT angiography[MeSH Terms] OR coronary computed tomographic angiography[MeSH Terms] OR CCTA[MeSH Terms] AND machine learning[AB] OR artificial intelligence[AB] OR AI[AB] OR deep learning [AB] OR learning algorithm[AB] ) |
| Elsevier | (Radiom*[ABTI]) AND (Coronary artery disease[ABTI] OR CAD[ABTI] OR Coronary Disease[ABTI] OR Coronary Atherosclerosis[ABTI] ) AND MACE[ABTI] OR major adverse cardiovascular events[ABTI] AND (CT[ABTI] OR coronary CT angiography[ABTI] OR coronary computed tomographic angiography[ABTI] OR CCTA[ABTI] AND machine learning[ABTI] OR artificial intelligence[ABTI] OR AI[ABTI] OR deep learning [ABTI] OR learning algorithm[ABTI]) |
| CNKI | (Radiom*[全文]) AND MACE[篇关摘] OR major adverse cardiovascular events[篇关摘] AND (coronary computed tomographic angiography[篇关摘] OR CCTA[篇关摘] AND machine learning[全文] OR artificial intelligence[全文] OR AI[全文] OR deep learning [全文] OR learning algorithm[全文]) |
| Abbreviations: | AB=abstract;ABTI=abstract and title  CNKI=China National Knowledge Infrastructure |
