# Supplementary material for: The Machine Learning Models in Major Cardiovascular Adverse Events Prediction Based on Coronary Computed Tomography Angiography: Systematic Review
Source: J Med Internet Res. 2025 Jun 13;27:e68872. doi: 10.2196/68872 (PMC12205263; doi:10.2196/68872)
Supplement: Multimedia Appendix 4 [file jmir_v27i1e68872_app4.docx]

Multimedia Appendix 4

**
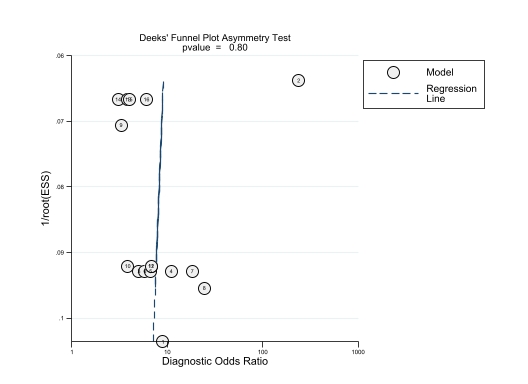

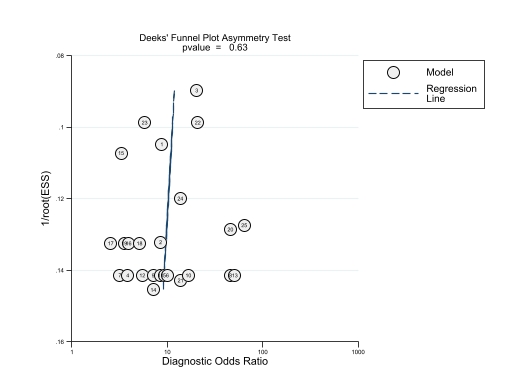
**

**Figure S1.** Publication bias (left-training set, right- testing set).

| Study | Image protocol quality | Multiple segmentations | Phantom study on all scanners | Imaging at multiple time points | Feature reduction or adjustment for multiple testing | Multivariable analysis with non radiomics features | Detect and discuss biological correlates | Cut-off analyses | Discrimination statistics | Calibration statistics | Prospective study registered in a trial database | Validation | Comparison to ‘gold standard’ | Potential clinical utility | Cost-effectiveness analysis | Open science and data | Total  Point |
| --- | --- | --- | --- | --- | --- | --- | --- | --- | --- | --- | --- | --- | --- | --- | --- | --- | --- |
| Feng,2022 | 1 | 1 | 0 | 0 | 3 | 0 | 0 | 1 | 2 | 0 | 0 | 3 | 2 | 0 | 0 | 2 | 15(41.67%) |
| Jing,2024 | 1 | 0 | 0 | 0 | 3 | 0 | 0 | 0 | 2 | 2 | 0 | 2 | 2 | 2 | 0 | 2 | 16(44.44%) |
| Li,2021 | 1 | 0 | 0 | 0 | 3 | 0 | 0 | 1 | 1 | 0 | 0 | 2 | 2 | 0 | 0 | 2 | 12(33.33%) |
| Militello,2023 | 1 | 1 | 0 | 0 | 3 | 1 | 1 | 0 | 2 | 2 | 0 | -5 | 2 | 0 | 0 | 4 | 12(33.33%) |
| Qin,2021 | 2 | 0 | 0 | 0 | 3 | 1 | 0 | 1 | 2 | 2 | 0 | 2 | 2 | 2 | 0 | 1 | 18(50.00%) |
| Wang,2023 | 1 | 1 | 1 | 0 | 3 | 0 | 0 | 1 | 2 | 2 | 0 | 4 | 2 | 2 | 0 | 4 | 23(63.89%) |
| Wang,2024 | 2 | 0 | 0 | 0 | 3 | 1 | 1 | 0 | 2 | 2 | 0 | 2 | 2 | 0 | 0 | 1 | 16(44.44%) |
| You,2023 | 1 | 1 | 0 | 0 | 3 | 1 | 1 | 0 | 2 | 2 | 0 | 2 | 2 | 2 | 0 | 4 | 21(58.33%) |
| Zhang,2024 | 1 | 0 | 0 | 0 | 3 | 1 | 0 | 1 | 2 | 1 | 0 | 2 | 2 | 2 | 0 | 3 | 18(50.00%) |
| Huang,2024 | 1 | 0 | 1 | 0 | 3 | 1 | 0 | 1 | 1 | 1 | 0 | 4 | 2 | 2 | 0 | 4 | 21(58.33%) |

**Figure S2.**  Radiomics quality score (RQS) per component of all included studies.


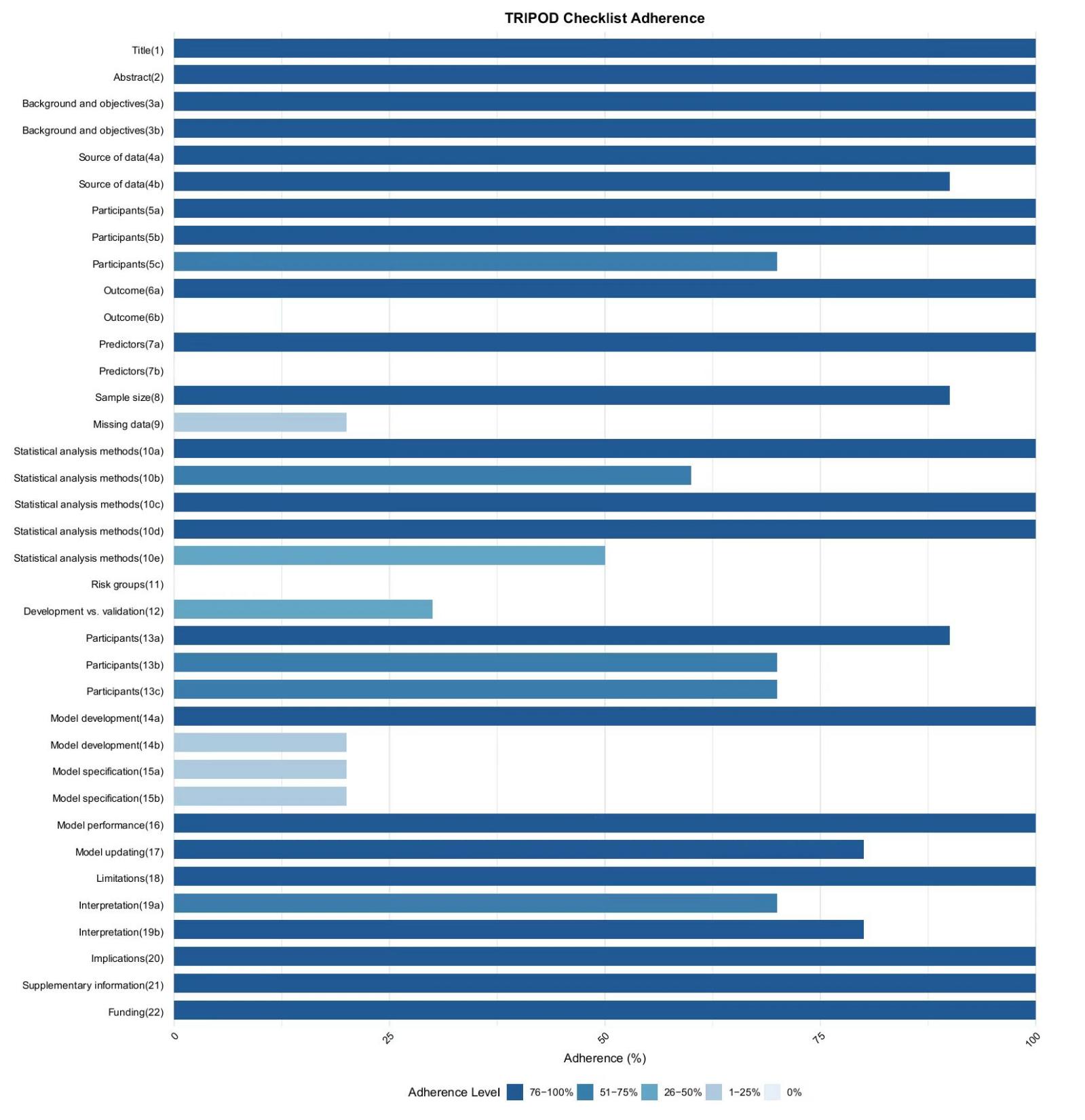


**Figure S3.** Overall adherence per TRIPOD item in testing or validation and training models.


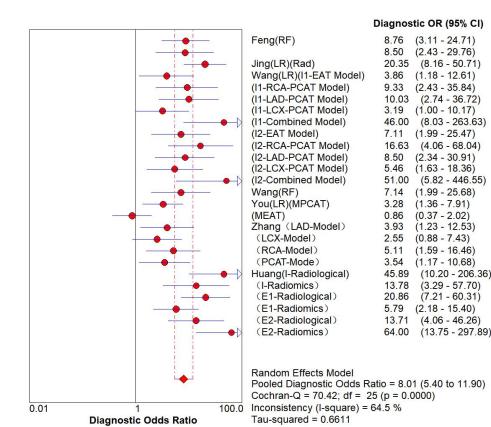

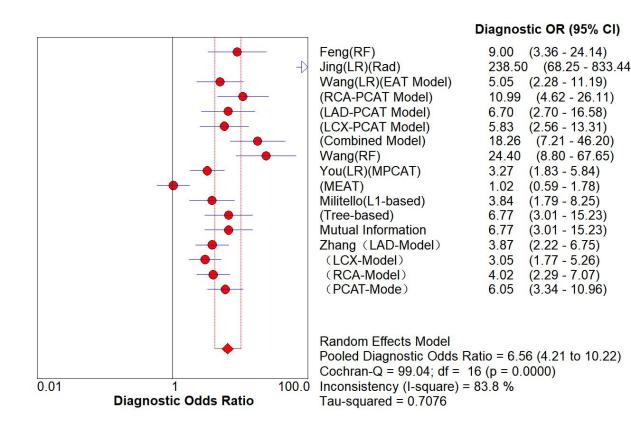


**Figure S4.** The pooled DOR (testing-training).


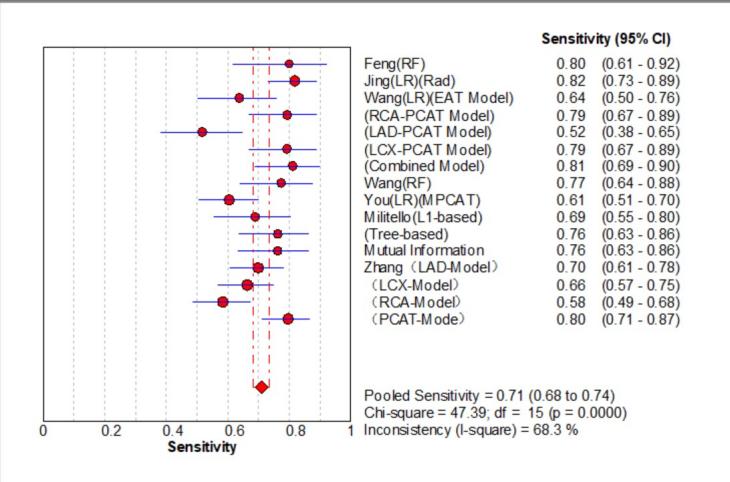

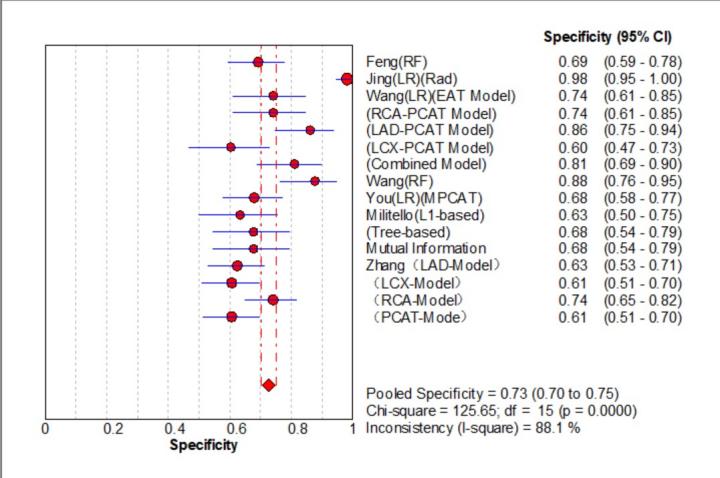


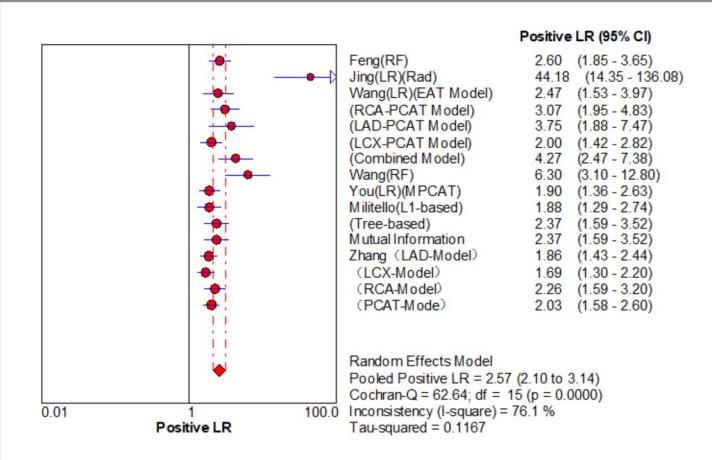

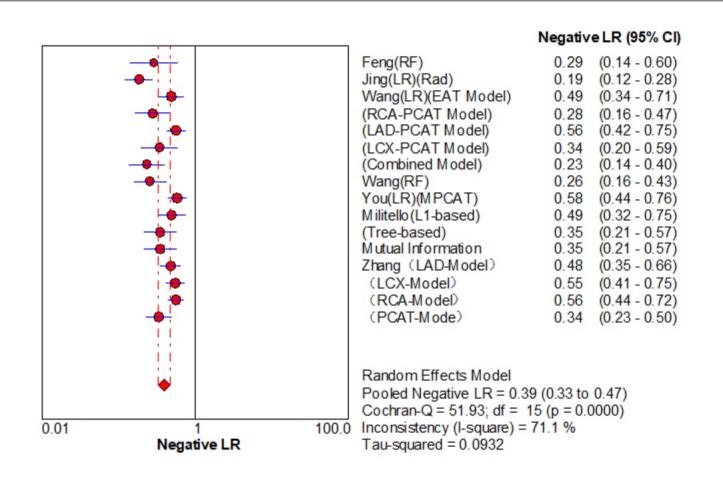


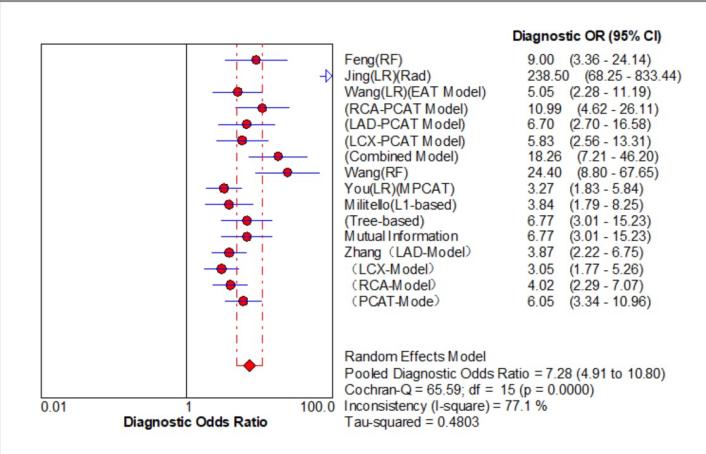


**Figure S5-S9.** Forest plots of ML models for predicting MACE in training set.


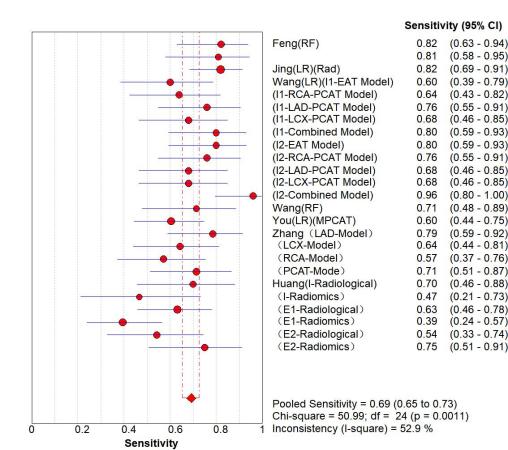

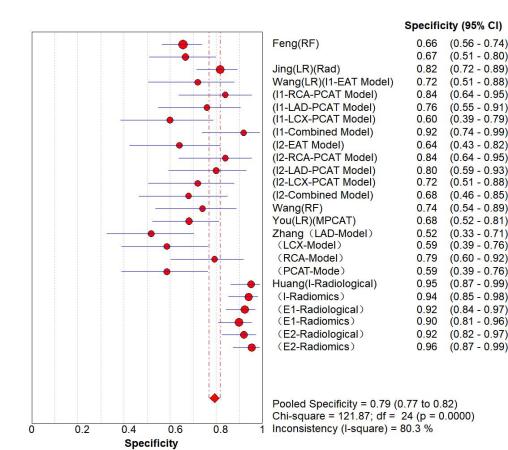


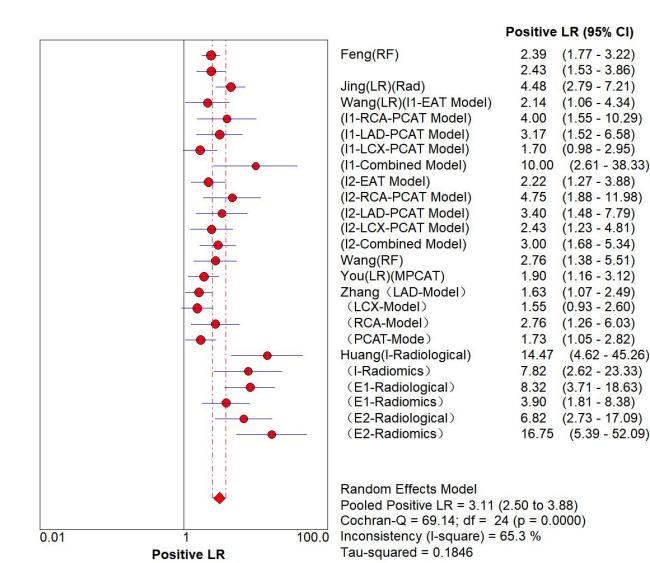

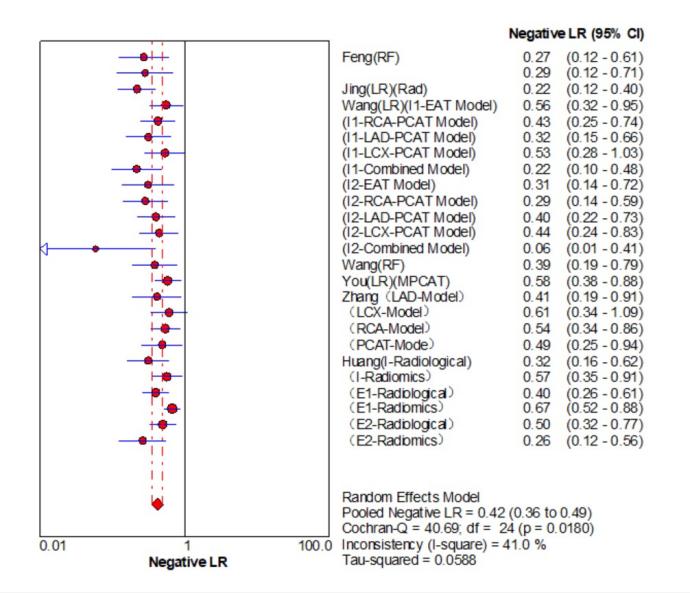


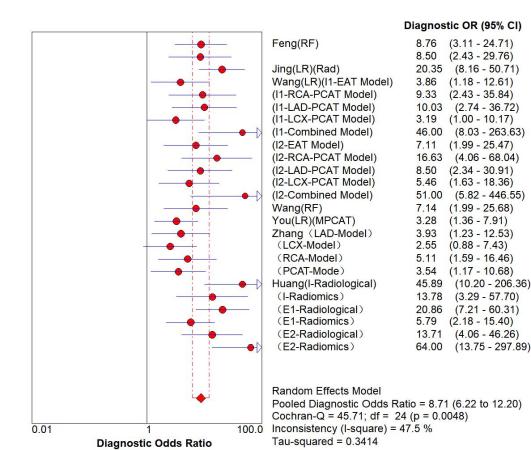


**Figure S10-S14.** Forest plots of ML models for predicting MACE in testing or validation set.


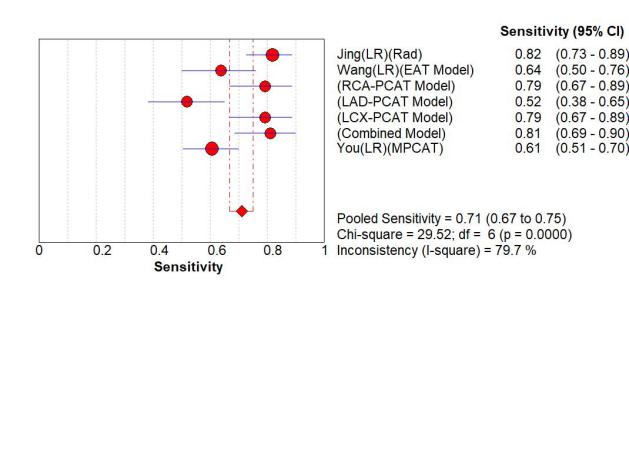

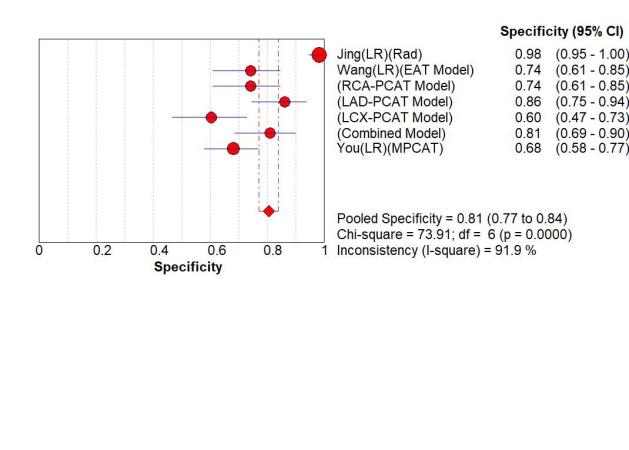


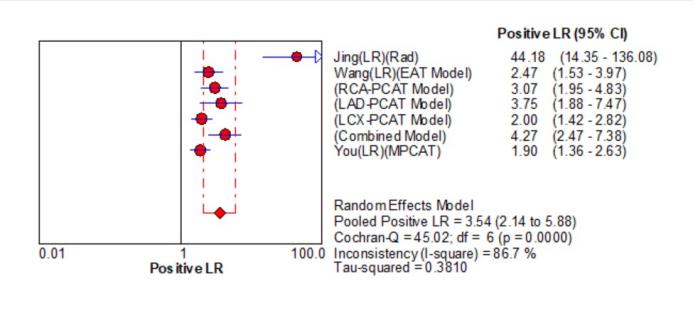

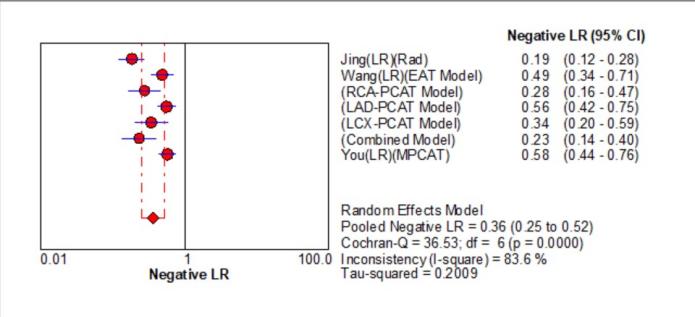


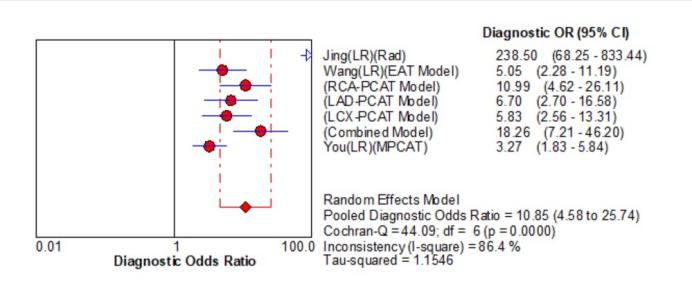

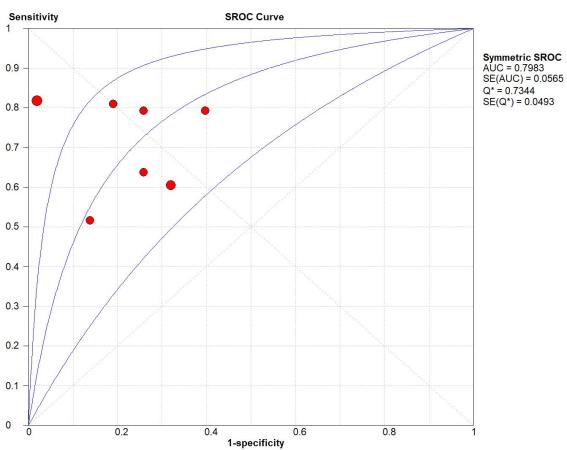


**Figure S15-S20.** Forest plots of **LR** models for predicting MACE in training set (subgroup analysis).


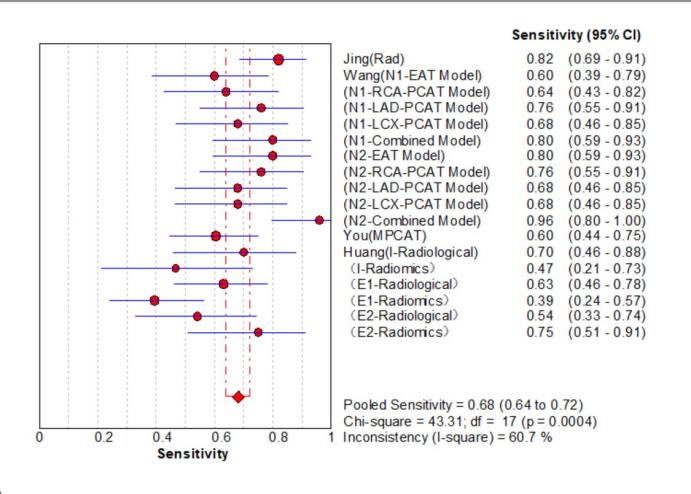

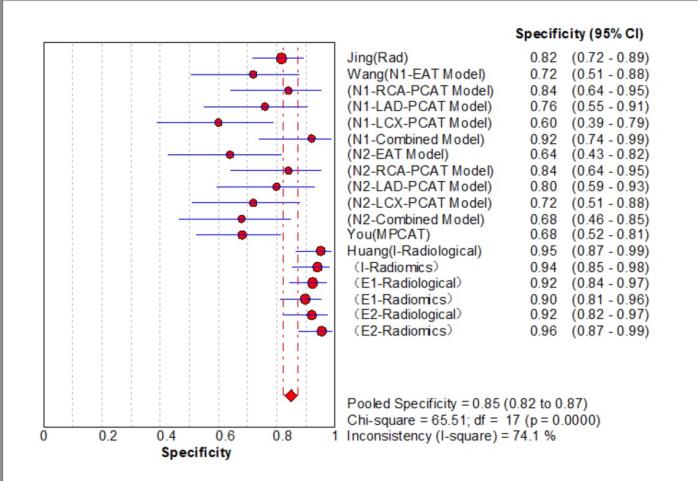

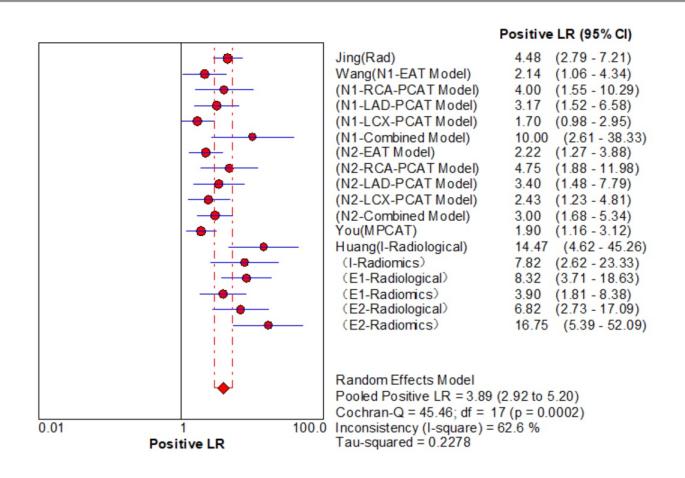

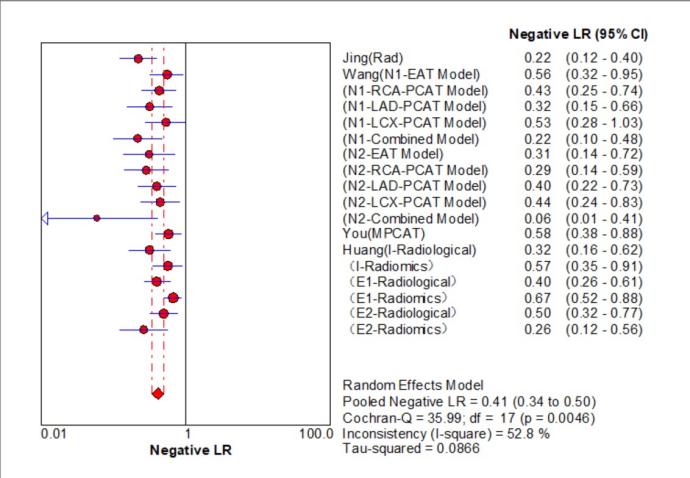


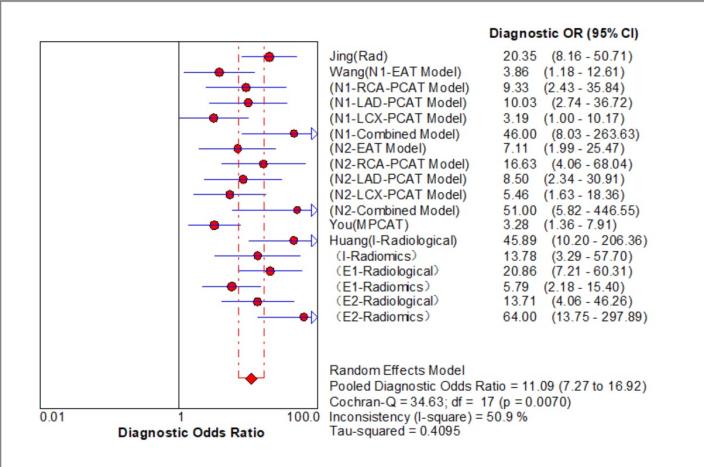

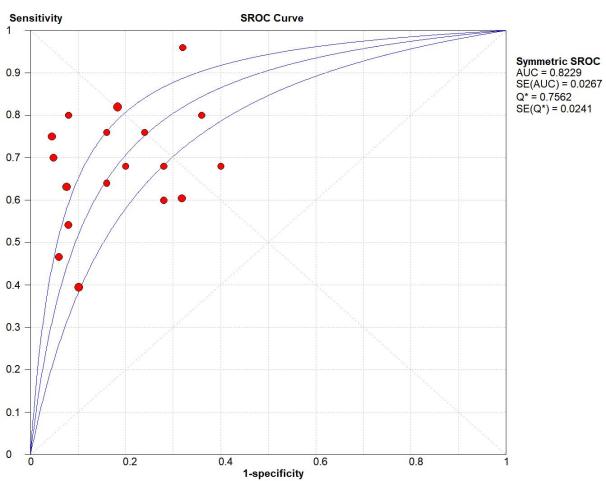


**Figure S21-S26.** Forest plots of **LR** models for predicting MACE in testing or validation set (subgroup analysis).


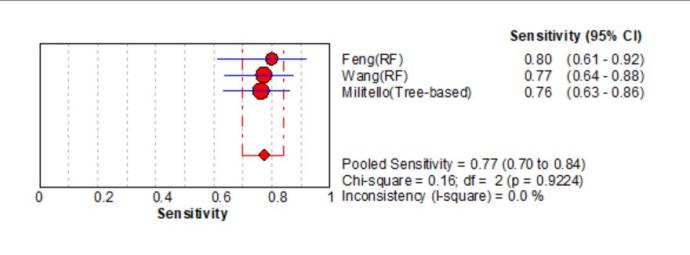

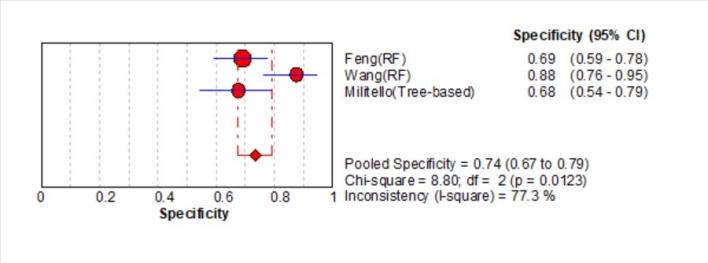


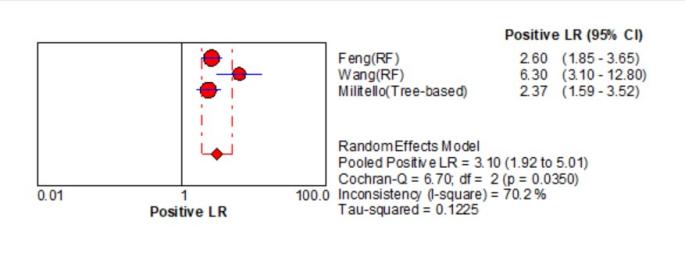

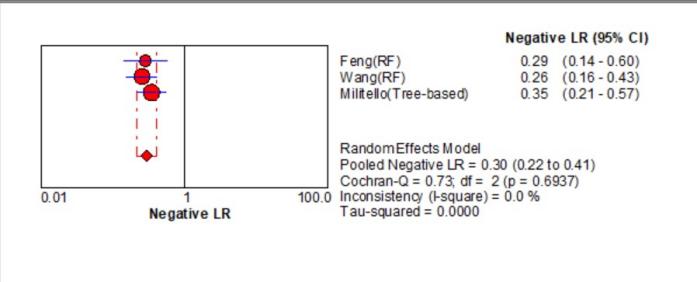


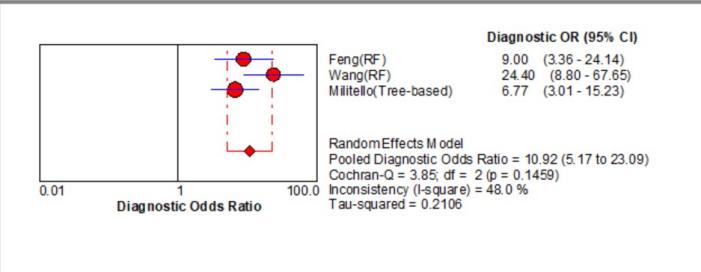

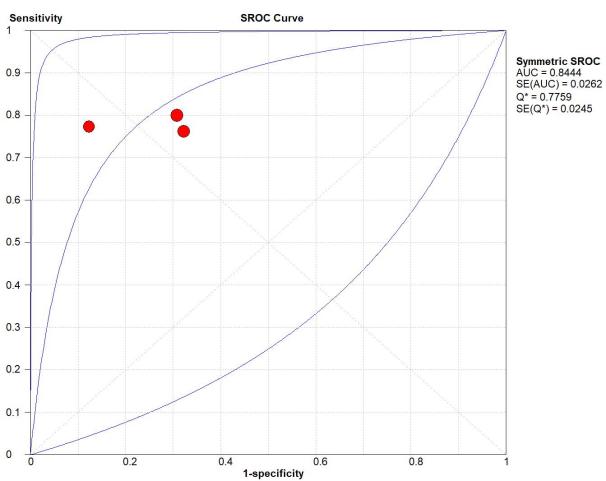


**Figure S27-S32.** Forest plots of **RF** models for predicting MACE in training set (subgroup analysis).

**
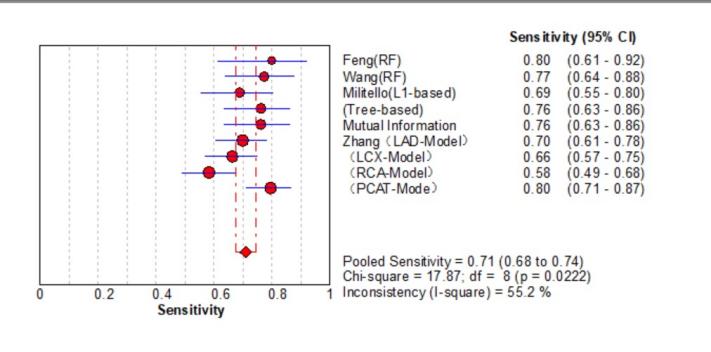

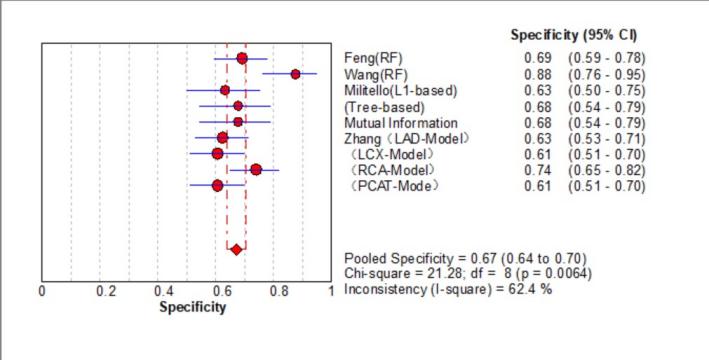
**

**
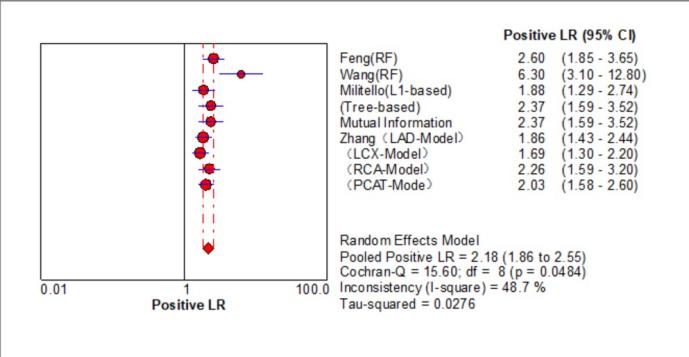

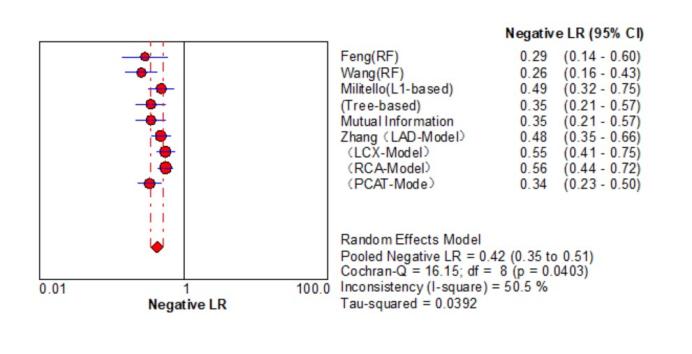
**

**
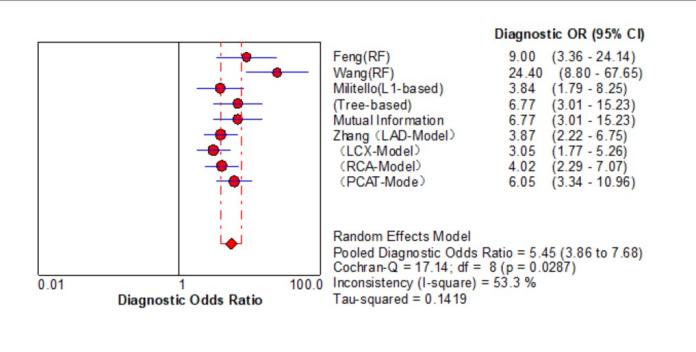
**
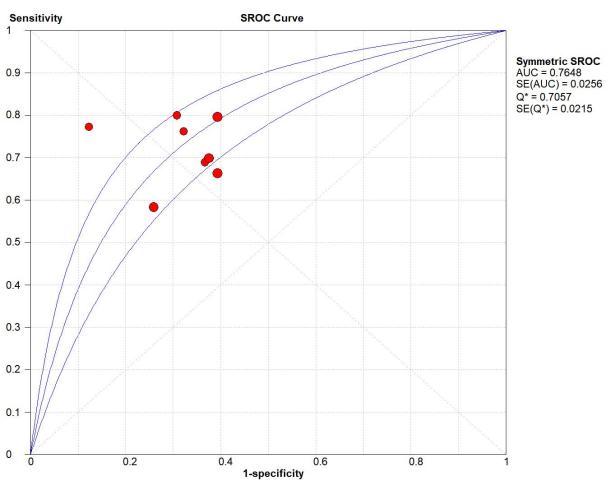


**Figure S33-S38.** Forest plots of **NLR** models for predicting MACE in training set (subgroup analysis).

**
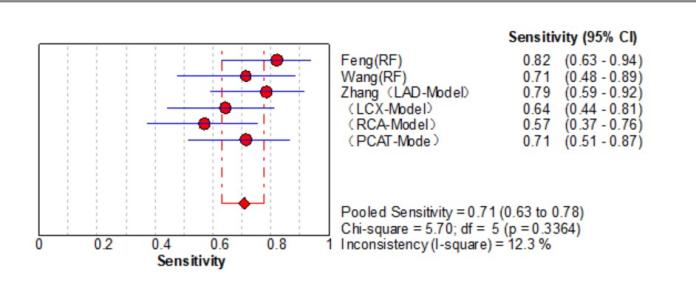

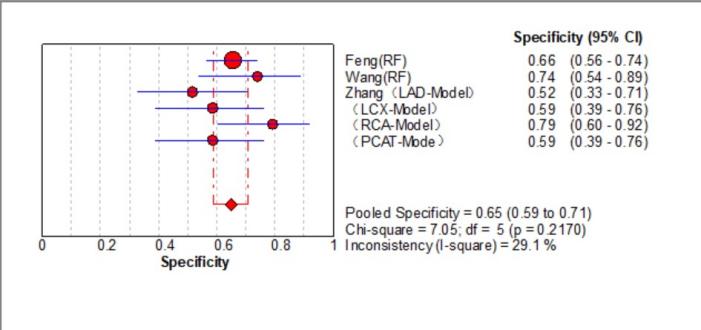
**

**
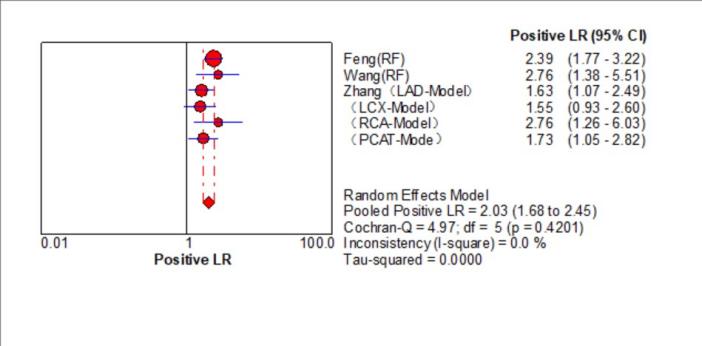

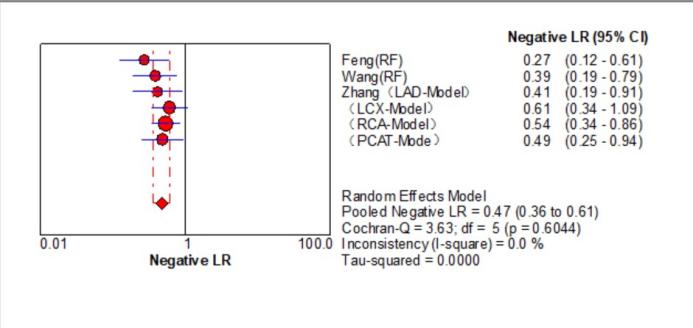
**

**
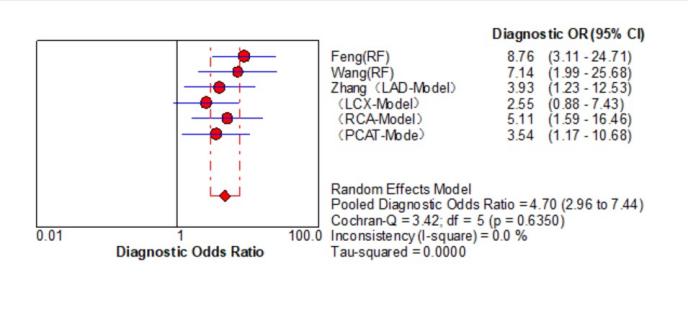
**
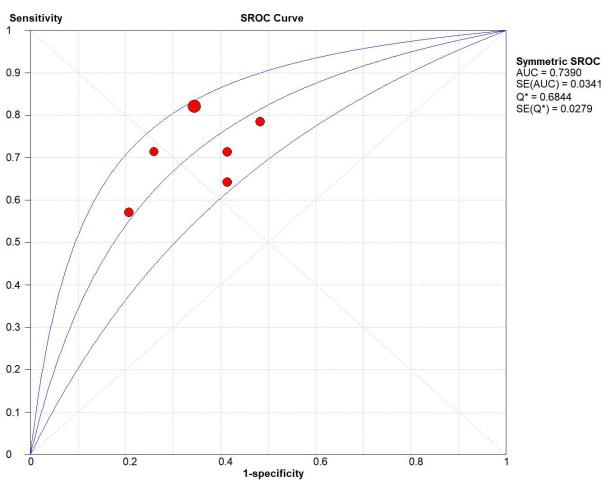


**Figure S39-S44.** Forest plots of **NLR** models for predicting MACE in testing or validation set (subgroup analysis).


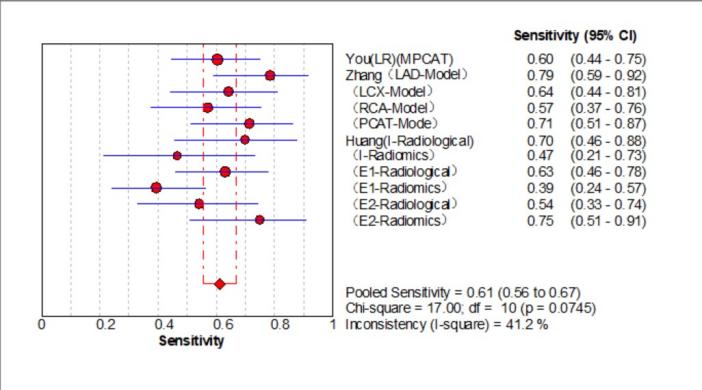

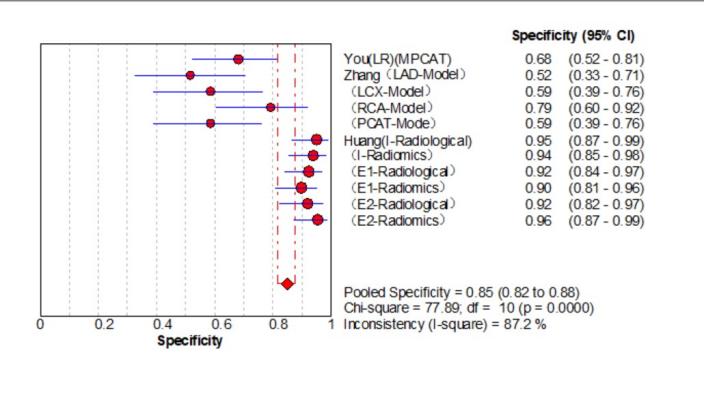


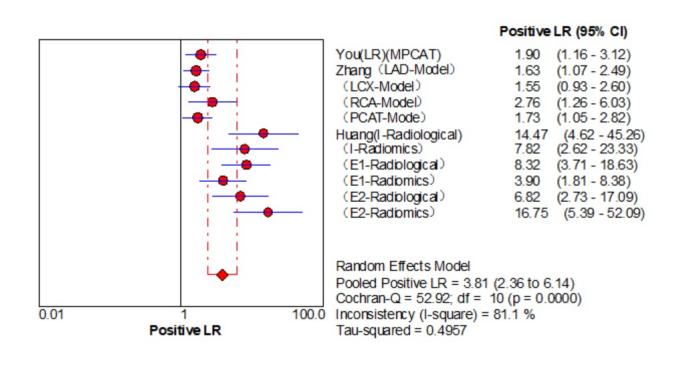

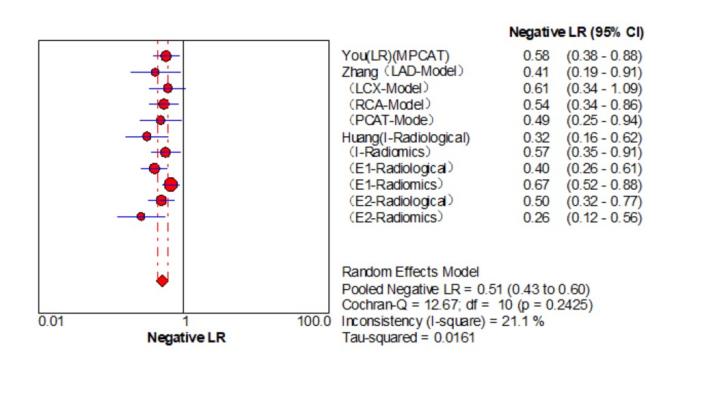


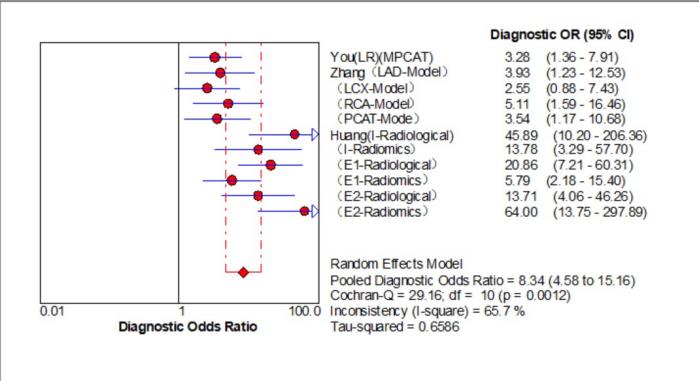

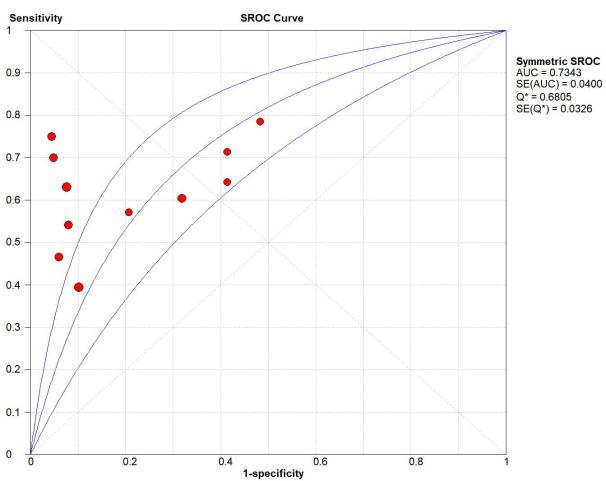


**Figure S45-S50.** Forest plots of ML models for MACE endpoints in testing or validation set (subgroup analysis).
